# Supplementary material for: Model-driven generation of artificial yeast promoters
Source: Nat Commun. 2020 Apr 30;11:2113. doi: 10.1038/s41467-020-15977-4 (PMC7192914; doi:10.1038/s41467-020-15977-4)
Supplement: Supplementary file 1 — Supplementary Information [file 41467_2020_15977_MOESM1_ESM.pdf]

Model-driven generation of artificial yeast promoters

Kotopka and Smolke

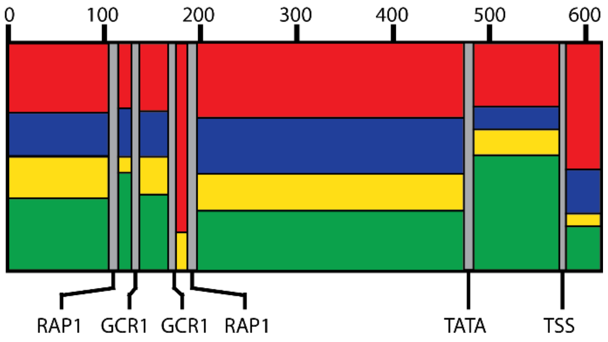

Supplementary Figure 1: Schematic of the wild-type P_{GPD} promoter. Locations of conserved motifs (gray) and spacer sequences in the wild-type P_{GPD} sequence; position scale appears across the top of the image, starting from the 5’ end of the sequence. Colored bars indicate the fractional base composition of each spacer sequence (red: A, blue: C, yellow: G, green: T). RAP1, GCR1: binding sites for transcription factors Rap1p, Gcr1p; TATA: TATA box motif; TSS: transcription start site.

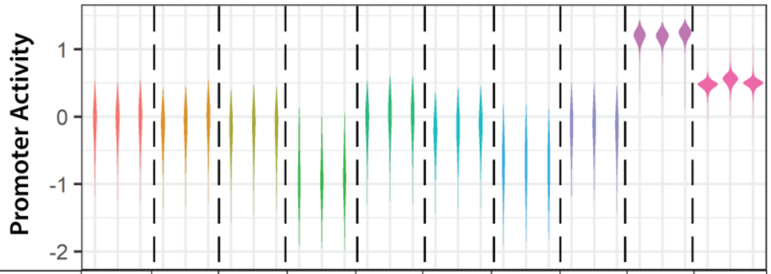

Construct	1	2	3	4	5	6	7	Final	pGPD	pTEF
RAP1 Len.	20,18	20,18	10,10	-	20,18	20,18	-	10,10		
TF Bias	+	-	-	-	+	+	-	-		
Int. Len.	37	37	37	37	74	37	74	74		
TATA Len.	18	18	18	18	18	10	10	10		

Supplementary Figure 2: Activity measurements in triplicate for P_{GPD} library candidates. Flow cytometry measurements of promoter activity, in triplicate, for each of eight designed P_{GPD} library candidates, with P_{GPD} and P_{TEF1}-expressing controls for comparison. Each group is assigned a different color as a guide for the eye. RAP1 Len.: length of first, second RAP1 motifs included; - if not present; TF Bias: use of wild-type (biased) or uniform base frequencies in the three spacer sequences between the TFBSes; Int. Len.: length of the internal spacer between the last TFBS and the TATA box; TATA Len.: length of the TATA motif (ten core bases or an additional 4 on each side).

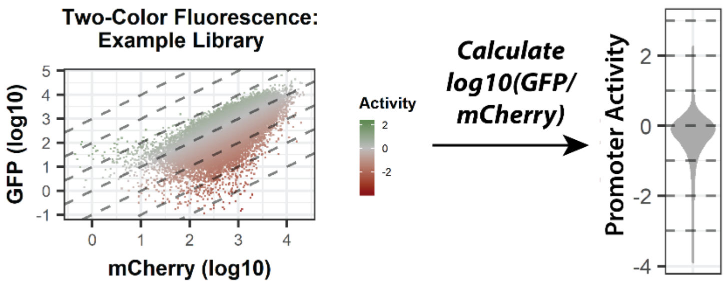

Supplementary Figure 3: Converting raw GFP and mCherry fluorescence in a library to a final measure of promoter activity. To extract a distribution of promoter activities (right) from flow cytometry data (left), the ratio of GFP to mCherry fluorescence was determined for each cell measured and expressed as a base-10 logarithm.

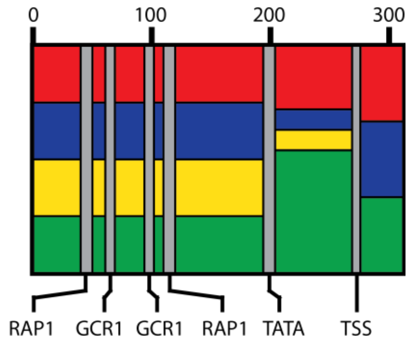

Supplementary Figure 4: Schematic of the final P_{GPD} construct (Final in Supp. Fig. 2). Color-coding is as in Supp. Fig. 1: red: A, blue: C, yellow: G, green: T, gray: conserved motif. RAP1, GCR1: binding sites for transcription factors Rap1p, Gcr1p; TATA: TATA box motif; TSS: transcription start site.

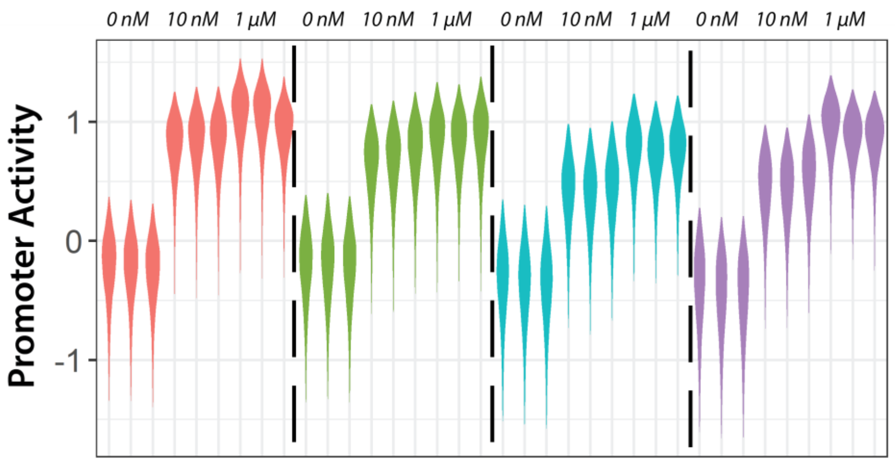

Construct	1	2	3	4
ZEV Sites	5	5	3	3
Int. Len.	37	74	74	37
TATA Len.	18	10	10	18

Supplementary Figure 5: Activity measurements in triplicate for P_{ZEV} library candidates. Flow cytometry measurements of promoter activity, in triplicate, for each of four designed P_{ZEV} library candidates. Each group is assigned a different color as a guide for the eye. For each construct (left to right), promoter activities with 0 nM, 10 nM, and 1 µM beta-estradiol induction are shown. ZEV sites: number of ZEV ATF binding sites included; Int. Len.: length of the internal spacer between the last TFBS and the TATA box; TATA Len.: length of the TATA motif (ten core bases or an additional 4 on each side).

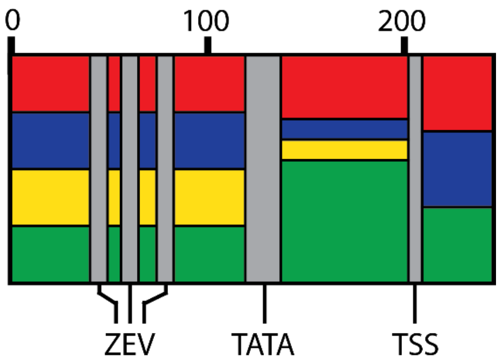

Supplementary Figure 6: Schematic of the final P_{ZEV} library. Color-coding is as in Supp. Fig. 1: red: A, blue: C, yellow: G, green: T, gray: conserved motif. ZEV: ZEV ATF binding sites; TATA: TATA box motif; TSS: transcription start site.

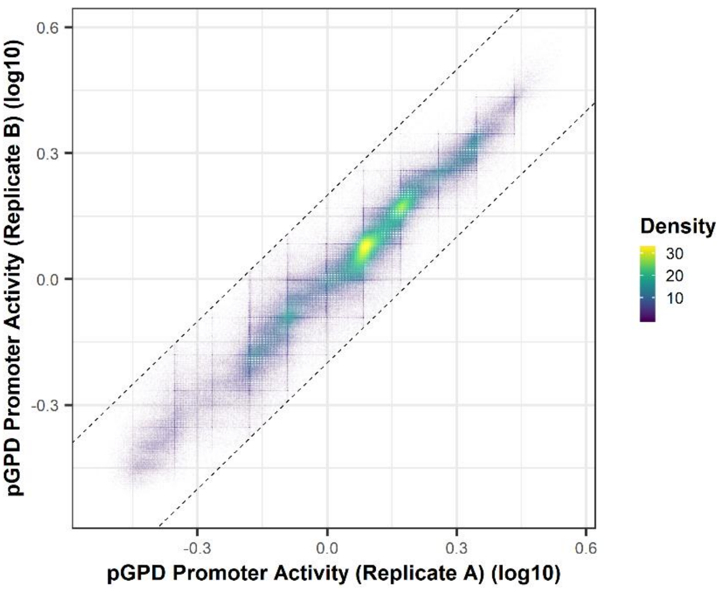

Supplementary Figure 7: Replicate measurements of P_{GPD} library sequences. Values are promoter activity measurements reported as the base 10 logarithm of GFP/mCherry ratio from FACS-seq data. Dashed lines: cutoff difference between replicates of 0.2, used to screen out discordant measurements as outliers. Only sequences for which at least 10 NextSeq reads were counted in each replicate were used in this analysis. Density: density of plotted points (arbitrary units).

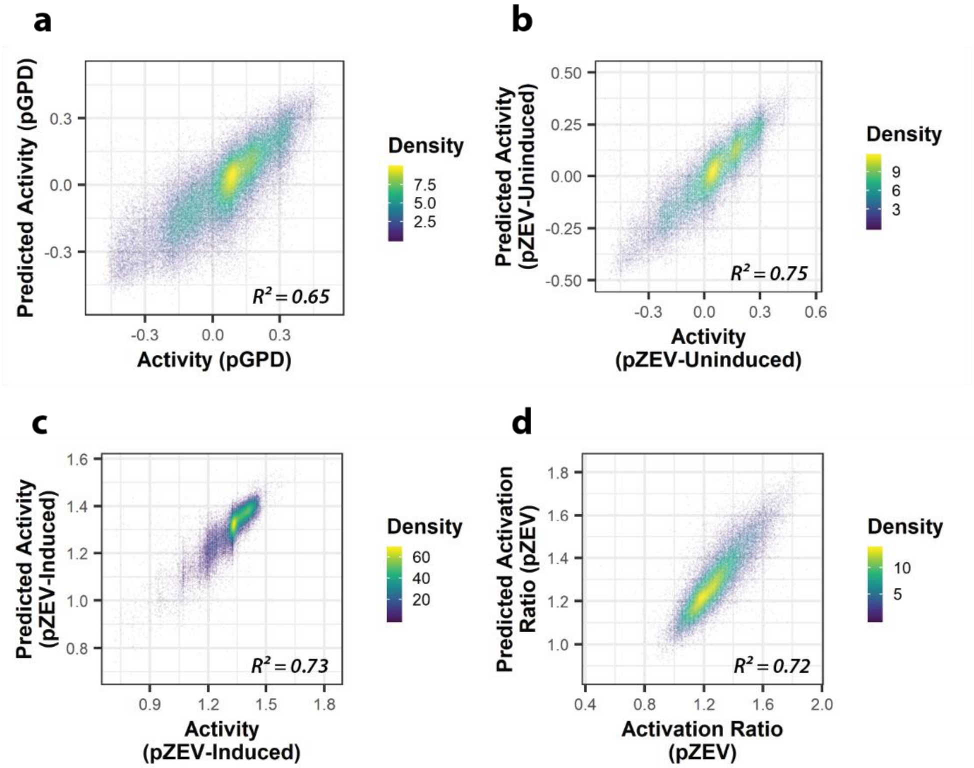

Supplementary Figure 8: Neural networks trained independently on P_{GPD} or P_{ZEV} data. Density: density of plotted points (arbitrary units).

a: Predicted promoter activities versus FACS-seq measurements for P_{GPD} sequences in the held-out test data.

b: Predicted promoter activities in the uninduced condition versus FACS-seq measurements for P_{ZEV} sequences in the held-out test data.

c: Predicted promoter activities in the induced condition versus FACS-seq measurements for P_{ZEV} sequences in the held-out test data.

d: Predicted activation ratios (ratio of predicted induced and uninduced promoter activities) for P_{ZEV} sequences in the held-out test data.

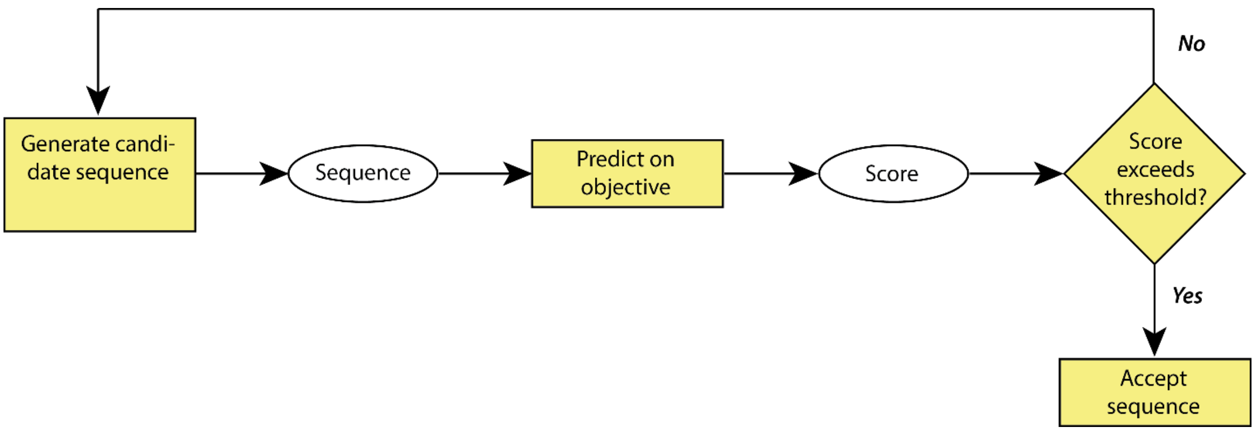

Supplementary Figure 9: Conceptual flowchart for model-guided promoter design by screening.

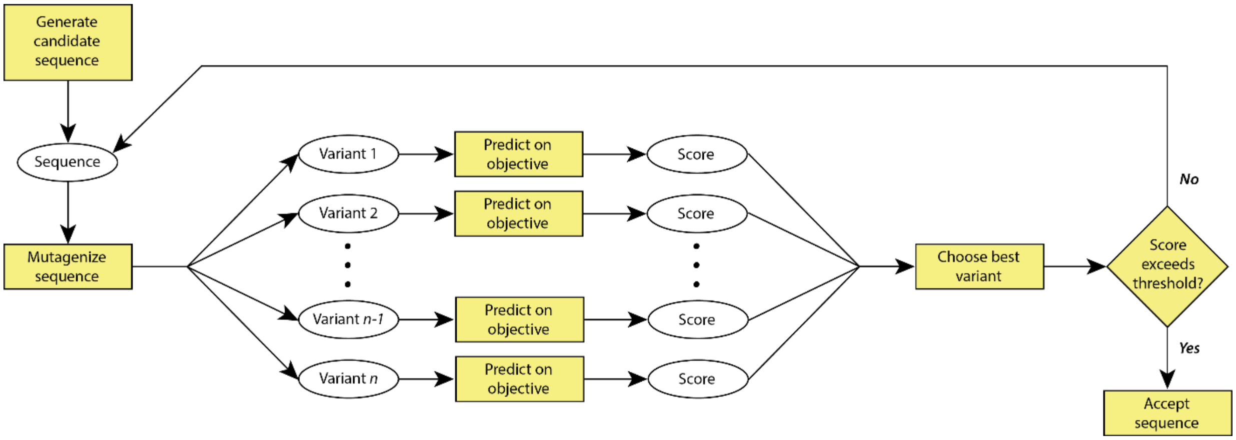

Supplementary Figure 10: Conceptual flowchart for model-guided promoter design by evolution.

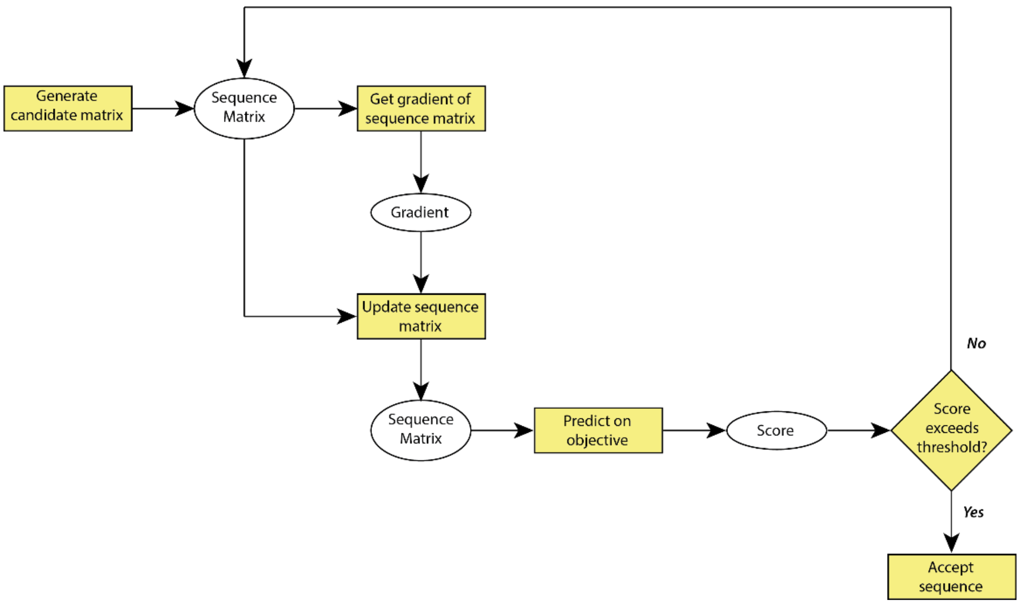

Supplementary Figure 11: Conceptual flowchart for model-guided promoter design by gradient ascent.

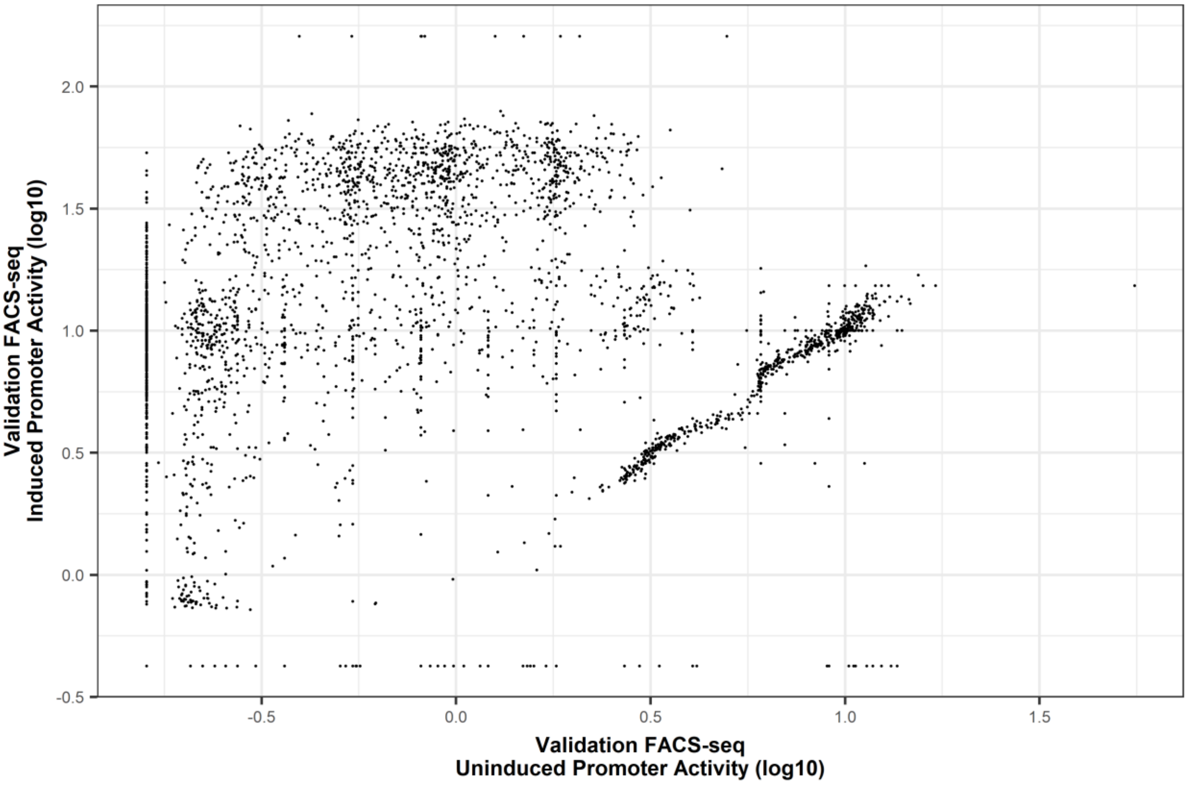

Supplementary Figure 12: Induced and uninduced promoter activities for all designed sequences measured in the validation FACS-seq. Only sequences for which at least 10 MiSeq reads were counted in each replicate were used in this analysis. Promoter activities shown here were transformed to a scale co-measureable with the results of individual promoter testing, using a linear model fit to promoter activities measured by FACS-seq and by individual testing for a set of promoters spanning a range of expression activities.

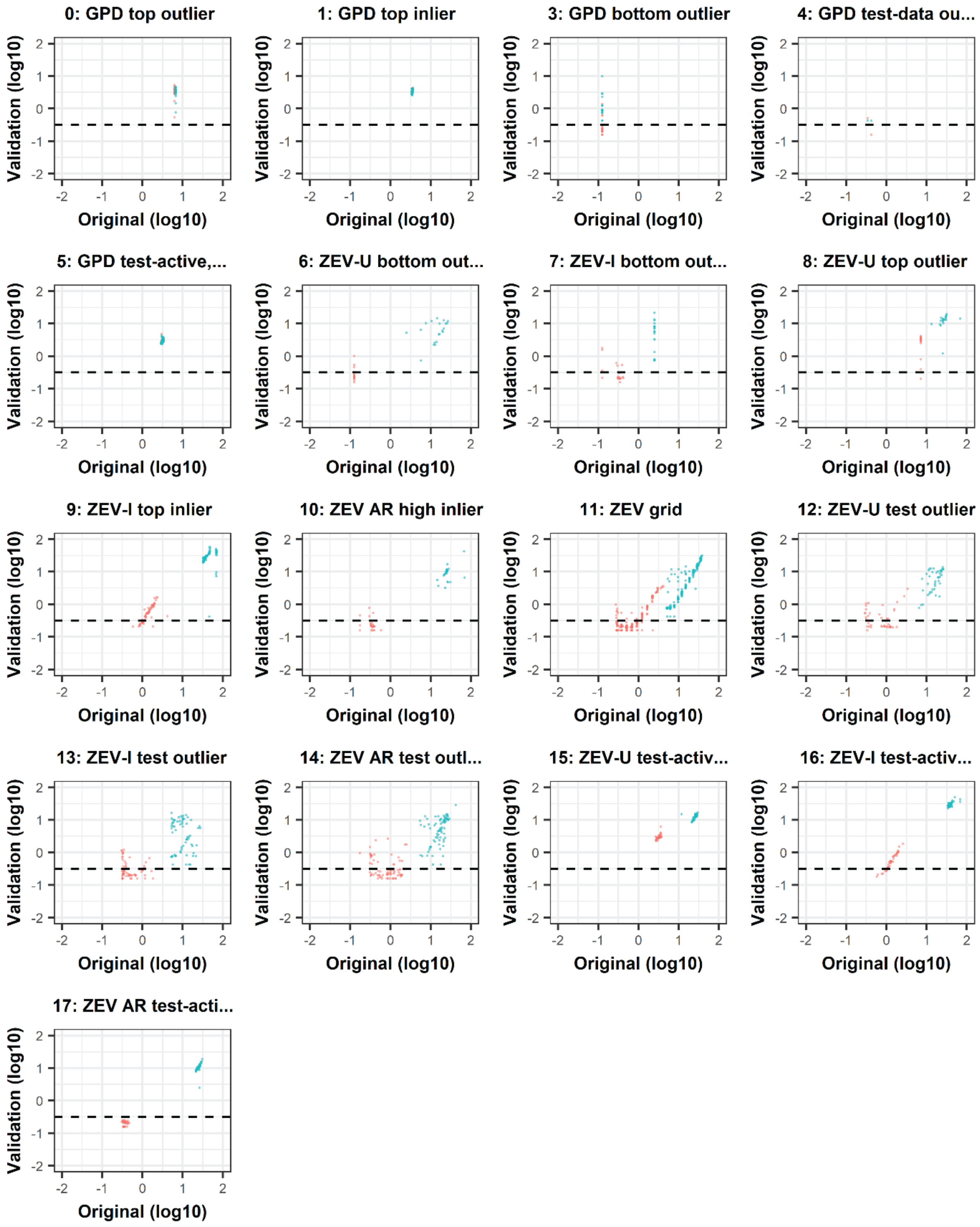

Supplementary Figure 13: Original and validation FACS-seq measurements of control promoters. For each set of control promoters tested in the validation FACS-seq experiment, promoter activities from the original experiment (the P_{GPD} or P_{ZEV} experiment, depending on the set) and from the validation experiment are plotted for condition A (red) and for condition B (blue).

(In P_{GPD}, A and B are strict replicates; in P_{ZEV}, these correspond respectively to the uninduced (no added beta-estradiol) and induced (1 µM beta-estradiol) conditions.) Figure titles correspond to promoter set names in Supplementary Table 3. Original: promoter activities measured in the original P_{GPD} or P_{ZEV} FACS-seq experiment; Validation: promoter activities measured in the validation FACS-seq experiment. Promoter activities shown here were transformed to a scale co-measureable with the results of individual promoter testing, using a linear model fit to promoter activities measured by FACS-seq and by individual testing for a set of promoters spanning a range of expression activities.

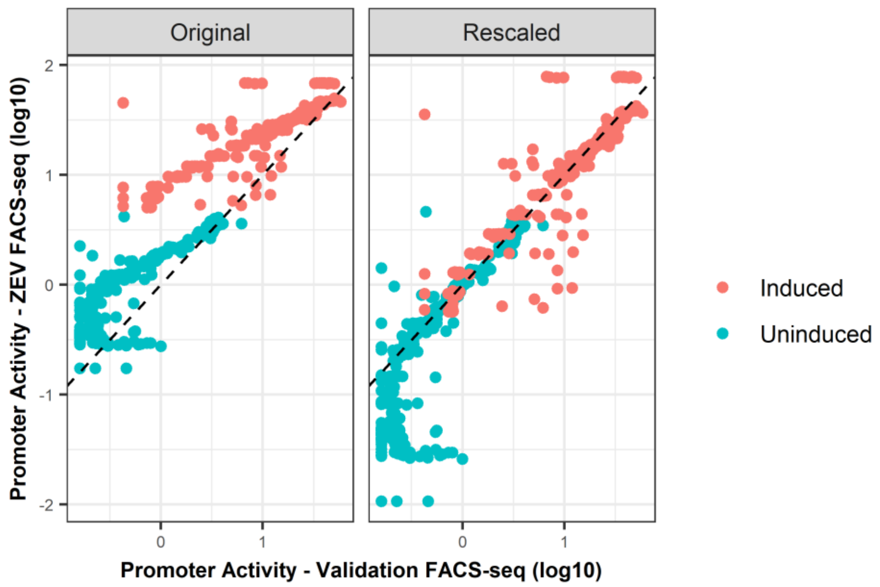

Supplementary Figure 14: Aligning P_{ZEV} data to validation FACS-seq results. Linear models were fit on the activities of shared sequences in the P_{ZEV} and validation FACS-seq experiments, allowing P_{ZEV} means to be recalculated to be co-measureable with sequences individually tested in FACS-seq. Uninduced: no added beta-estradiol. Induced: 1 µM beta-estradiol.

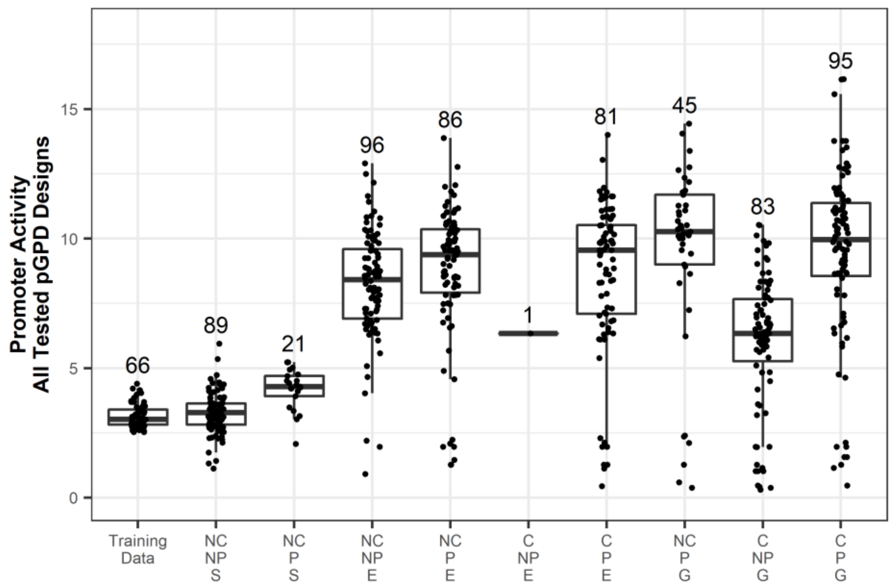

Supplementary Figure 15: Promoter activity for all P_{GPD} sequences measured in the design validation FACS-seq. Boxes represent interquartile ranges; the bar within each box indicates the median. Whiskers extend to the furthest observation within 1.5 interquartile ranges of the nearest box edge. Numbers over boxplots indicate the number of sequences measured in FACS-seq in each promoter set. Training Data: highly active sequences from the initial P_{GPD} FACS-seq, as in Fig. 3a; C/NC: GC constraint/no GC constraint; P/NP: extrapolation penalty/no extrapolation penalty; S, E, G: screening, evolution, or gradient ascent design strategy, respectively. Promoter activities shown here were transformed to a scale co-measureable with the results of individual promoter testing, using a linear model fit to promoter activities measured by FACS-seq and by individual testing for a set of promoters spanning a range of expression activities. Source data are provided as a Source Data file.

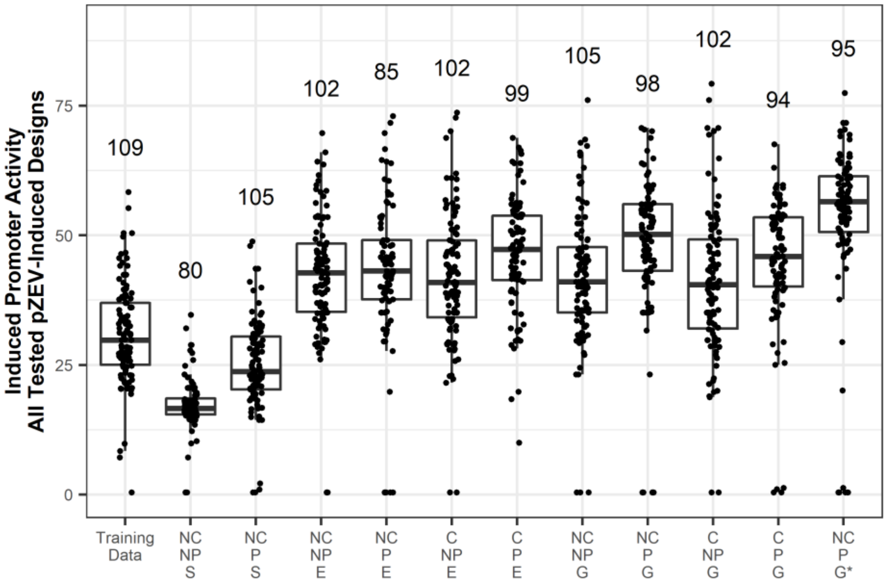

Supplementary Figure 16: Promoter activity for all P_{ZEV}-Induced sequences measured in the design validation FACS-seq. Boxes represent interquartile ranges; the bar within each box indicates the median. Whiskers extend to the furthest observation within 1.5 interquartile ranges of the nearest box edge. Numbers over boxplots indicate the number of sequences measured in FACS-seq in each promoter set. Training Data: sequences with high P_{ZEV}-Induced activity from the initial P_{ZEV} FACS-seq, as in Fig. 3b; C/NC: GC constraint/no GC constraint; P/NP: extrapolation penalty/no extrapolation penalty; S, E, G: screening, evolution, or gradient ascent design strategy, respectively. G*: P_{ZEV}-Induced promoter set generated using the gradient approach, with an elevated target threshold set relative to other designs. Promoter activities shown here were transformed to a scale co-measureable with the results of individual promoter testing, using a linear model fit to promoter activities measured by FACS-seq and by individual testing for a set of promoters spanning a range of expression activities. Source data are provided as a Source Data file.

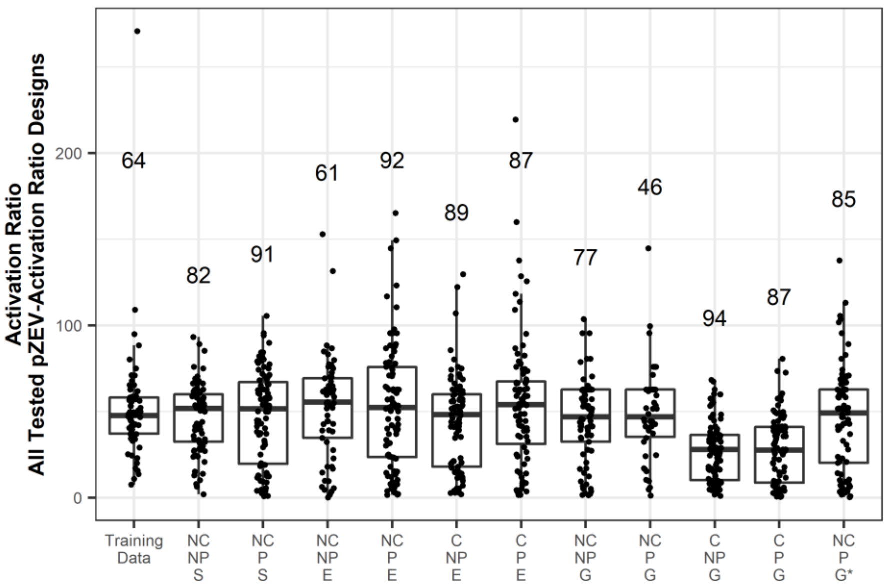

Supplementary Figure 17: Promoter activity for all P_{ZEV}-Activation Ratio sequences measured in the design validation FACS-seq. Boxes represent interquartile ranges; the bar within each box indicates the median. Whiskers extend to the furthest observation within 1.5 interquartile ranges of the nearest box edge. Numbers over boxplots indicate the number of sequences measured in FACS-seq in each promoter set. Training Data: sequences with high activation ratios from the initial P_{ZEV} FACS-seq, as in Fig. 3c; C/NC: GC constraint/no GC constraint; P/NP: extrapolation penalty/no extrapolation penalty; S, E, G: screening, evolution, or gradient ascent design strategy, respectively. G*: P_{ZEV}-Activation Ratio promoter set generated using the gradient approach, with an elevated target threshold set relative to other designs. Promoter activities shown here were transformed to a scale co-measureable with the results of individual promoter testing, using a linear model fit to promoter activities measured by FACS-seq and by individual testing for a set of promoters spanning a range of expression activities. Source data are provided as a Source Data file.

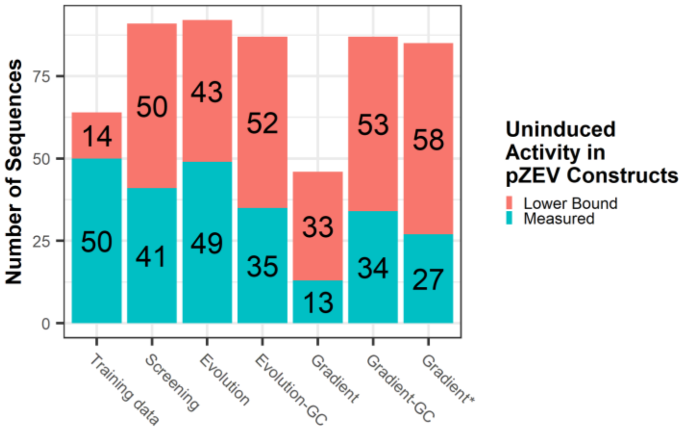

Supplementary Figure 18: Off-scale measurements of uninduced promoter activity for P_{ZEV}-Activation Ratio designs in the validation FACS-seq. Sequence sets displayed in Fig. 3c, indicating the number of sequences in each set which were quantitated in the experiment (Measured) or which fell entirely in the lowest-activity bin during FACS-seq (Lower Bound). Numbers on bars indicate the number of sequences measured in the validation FACS-seq which fell into each category, for each sequence set. All displayed promoter sets were generated using the extrapolation penalty. Source data are provided as a Source Data file.

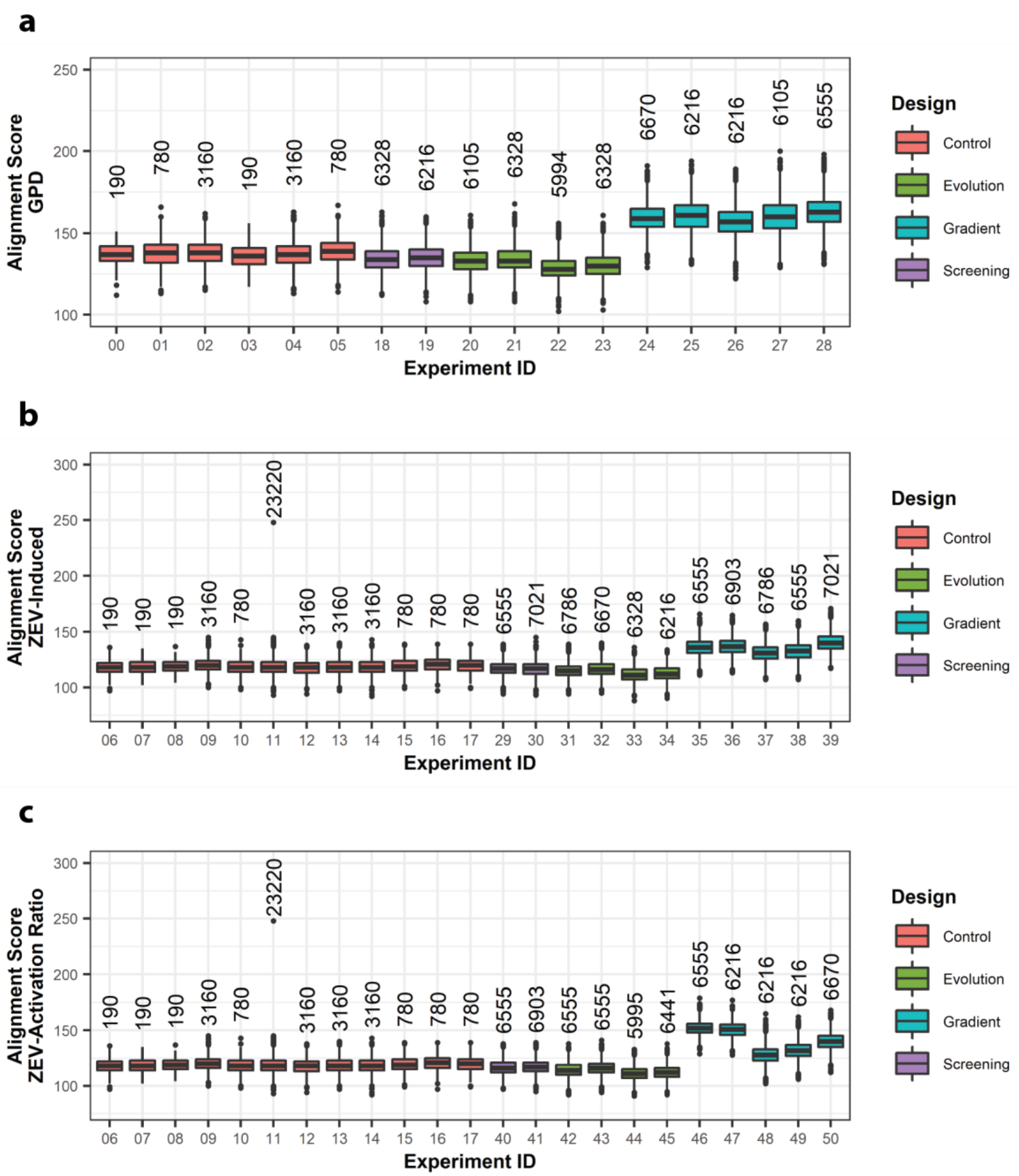

Supplementary Figure 19: Pairwise alignment distances within control and designed promoter sets. Needleman-Wunsch alignment distances were calculated between all possible sequence pairs in each control and each designed promoter set to investigate variations in sequence diversity from set to set. x-axis: Experiment ID identifying each promoter set, as in Supplementary Tables 2 and 3. Boxes represent interquartile ranges; the bar within each box indicates the median. Whiskers extend to the furthest observation within 1.5 interquartile ranges of the nearest box edge. Numbers over boxplots indicate the number of pairwise alignment distances calculated for each promoter set.

a: P_{GPD} promoter sets (controls and designs).

b: All P_{ZEV} control sets, and P_{ZEV}-Induced design sets.

c: All P_{ZEV} control sets, and P_{ZEV}-Activation Ratio design sets.

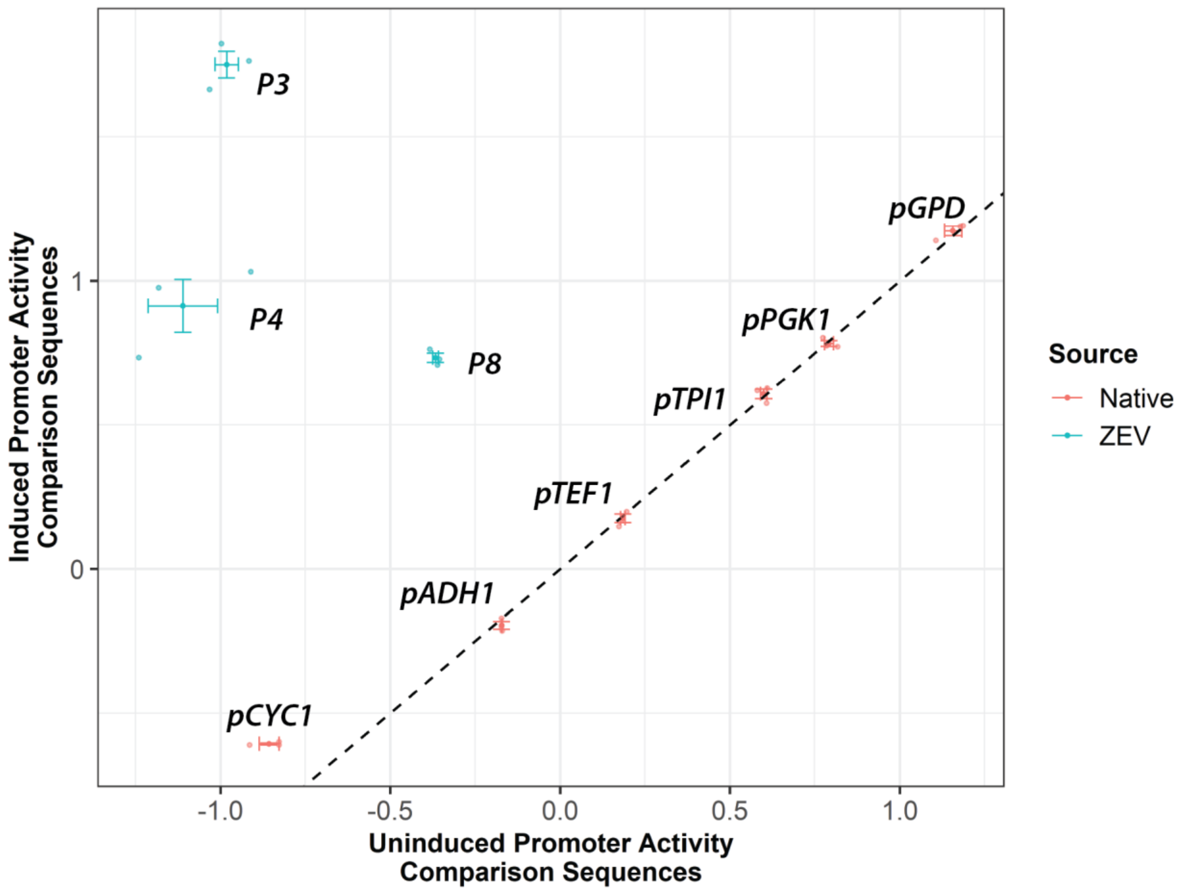

Supplementary Figure 20: Measured promoter activities in individual flow cytometry testing of comparison sequences, with and without induction with 1 μM beta-estradiol. Sequence names are as in Supplementary Tables 4 and 5. Error bars represent the standard error of the mean (n = 3 biologically independent samples). Source data are provided as a Source Data file.

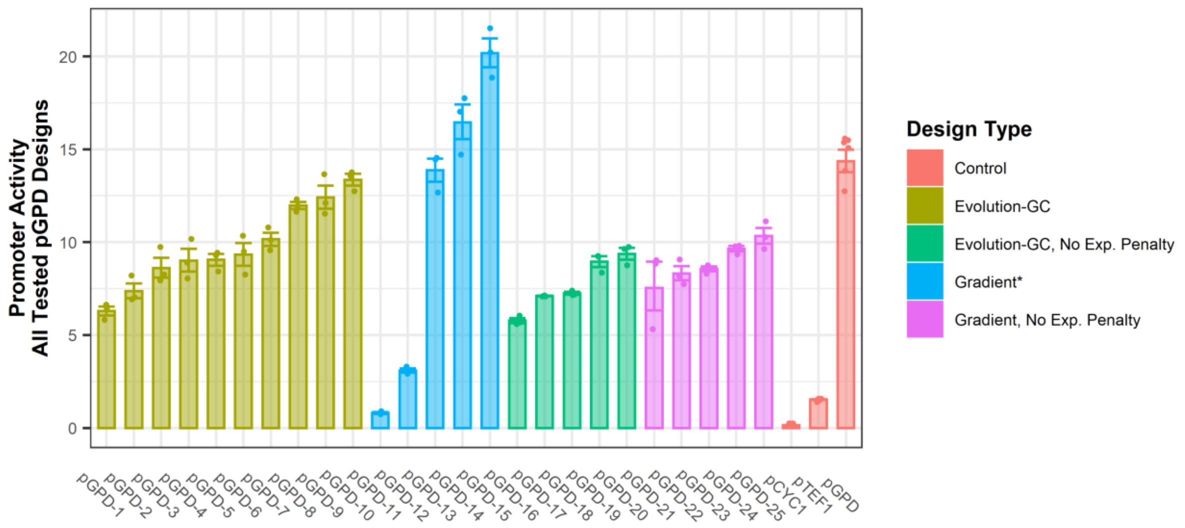

Supplementary Figure 21: Individually measured promoter activities determined by flow cytometry for selected P_{GPD} designs and for control sequences (Control). Evolution-GC: randomly chosen sequences from the selected P_{GPD} promoter set designed using the evolution strategy and the GC constraint, applying the extrapolation penalty; Evolution-GC, No Exp. Penalty: randomly chosen sequences from the P_{GPD} set designed as for Evolution-GC, but without the extrapolation penalty; Gradient*: randomly chosen sequences from the promoter set designed using the gradient strategy with an elevated threshold for selection; Gradient: randomly chosen sequences from the promoter set designed using the gradient strategy, without the extrapolation penalty, and at the selection threshold used for other evolution and gradient designs. The Evolution-GC and Gradient* sets also appear in Figure 4b. Sequence names are as in Supplementary Tables 4 and 5. Bars and error bars represent the mean and standard error of the mean (n = 3 biologically independent samples) for the original log-scale measurements, converted to linear scale. Source data are provided as a Source Data file.

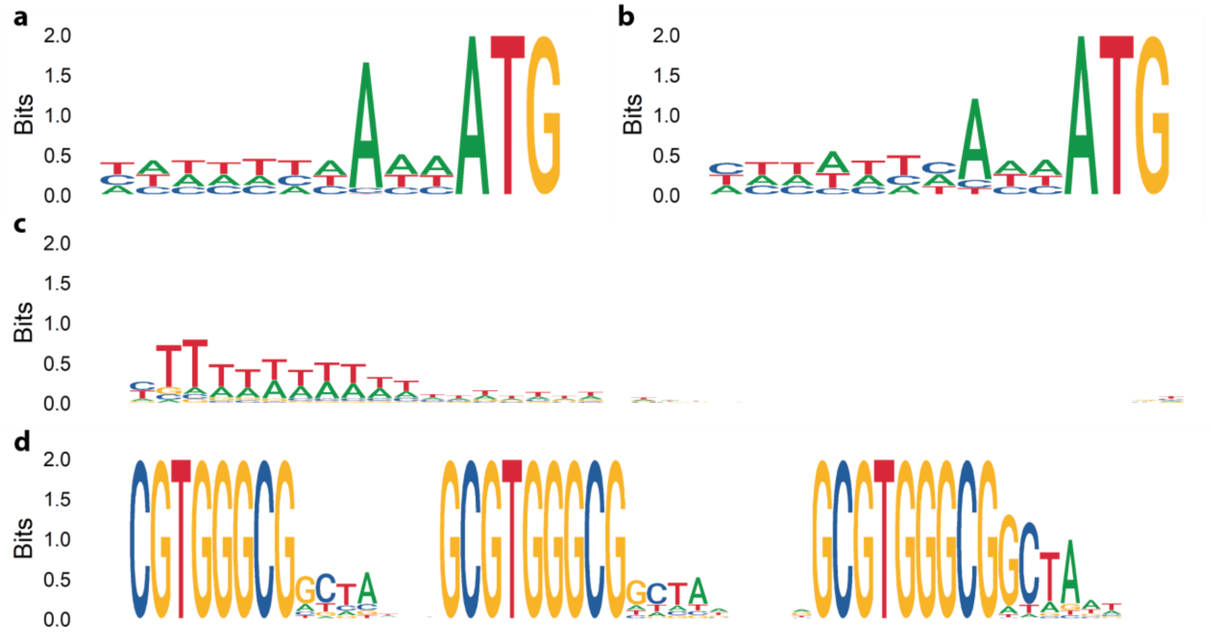

Supplementary Figure 22: Sequence logos for promoter sets characterized *in silico*. a: Sequence logo for 3’ region of P_{ZEV}-Induced design set. **b:** Sequence logo for 3’ region of P_{ZEV}-Activation Ratio design set. **c:** P_{ZEV}-Induced design set, upstream spacer (bases 1-40). **d:** P_{ZEV}-Activation Ratio design set, ZEV ATF binding site context (bases 42-92).

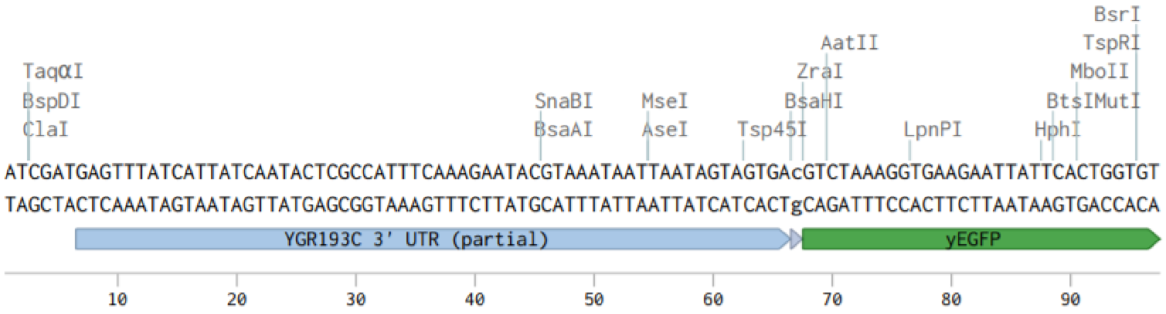

Supplementary Figure 23: Insertion context of the backbone plasmid pCS4306. Libraries and promoters are inserted into the ZraI cut site, as described in the main text.

a

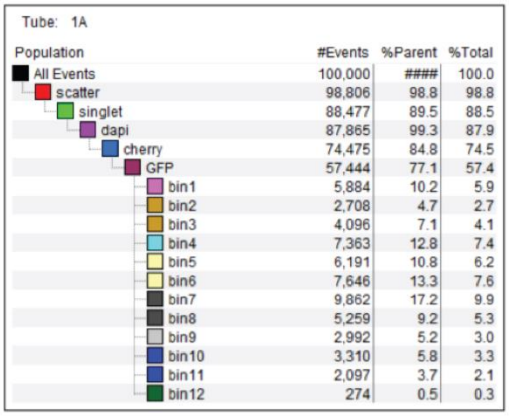

Tube: 1A

Population	#Events	%Parent	%Total
All Events	100,000	####	100.0
scatter	98,806	98.8	98.8
singlet	88,477	89.5	88.5
dapi	87,865	99.3	87.9
cherry	74,475	84.8	74.5
GFP	57,444	77.1	57.4
bin1	5,884	10.2	5.9
bin2	2,708	4.7	2.7
bin3	4,096	7.1	4.1
bin4	7,363	12.8	7.4
bin5	6,191	10.8	6.2
bin6	7,646	13.3	7.6
bin7	9,862	17.2	9.9
bin8	5,259	9.2	5.3
bin9	2,992	5.2	3.0
bin10	3,310	5.8	3.3
bin11	2,097	3.7	2.1
bin12	274	0.5	0.3

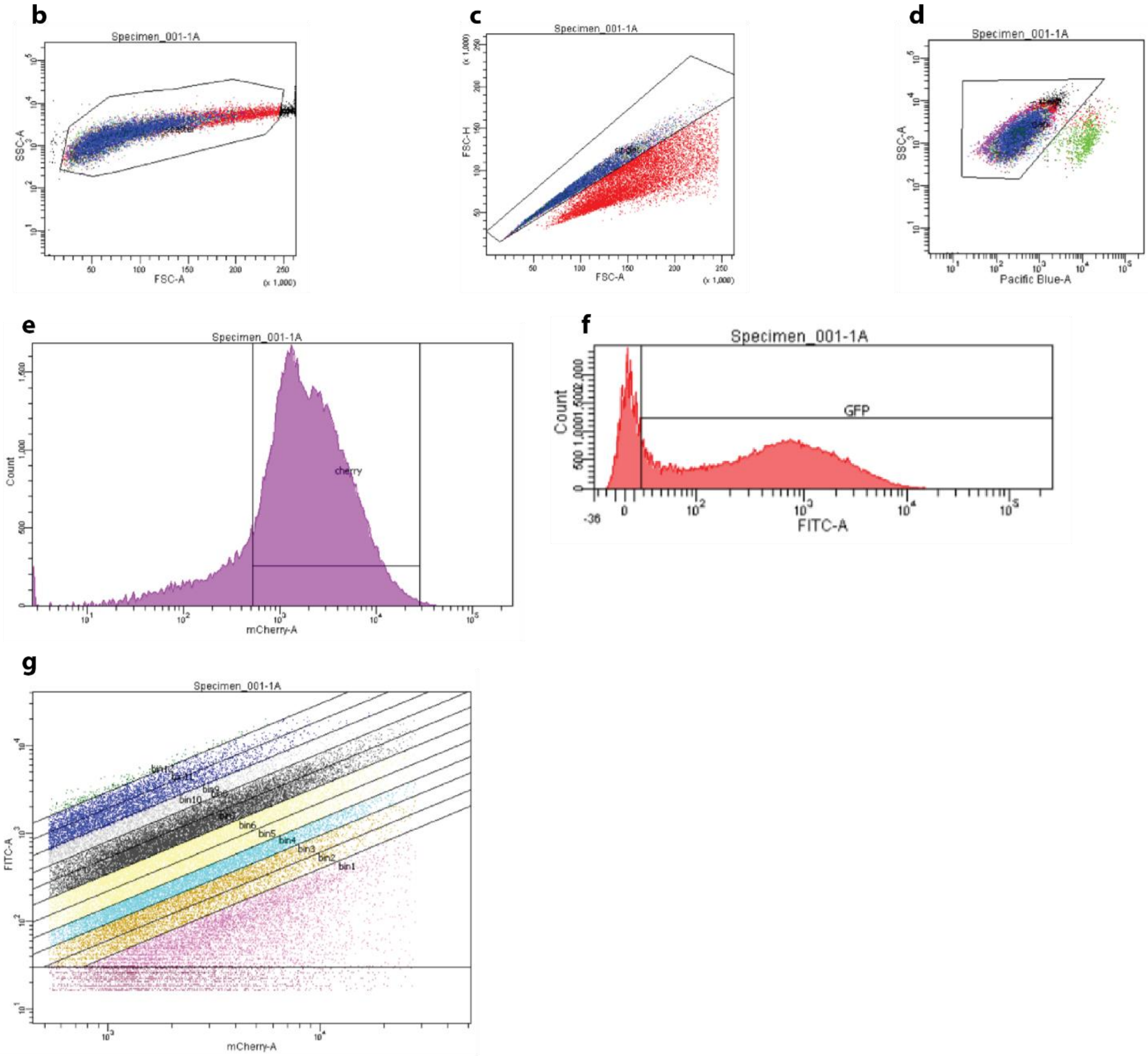

Supplementary Figure 24: FACS-seq gating strategy. a: Hierarchical view of gates used in FACS-seq sorts. **b:** FSC-A/SSC-A scatter gate. **c:** FSC-A/FSC-H singlet gate. **d:** Pacific Blue-A/SSC-A DAPI gate. **e:** mCherry activity gate. **f:** GFP activity gate. **g:** Twelve-bin gating strategy for final FACS sort.

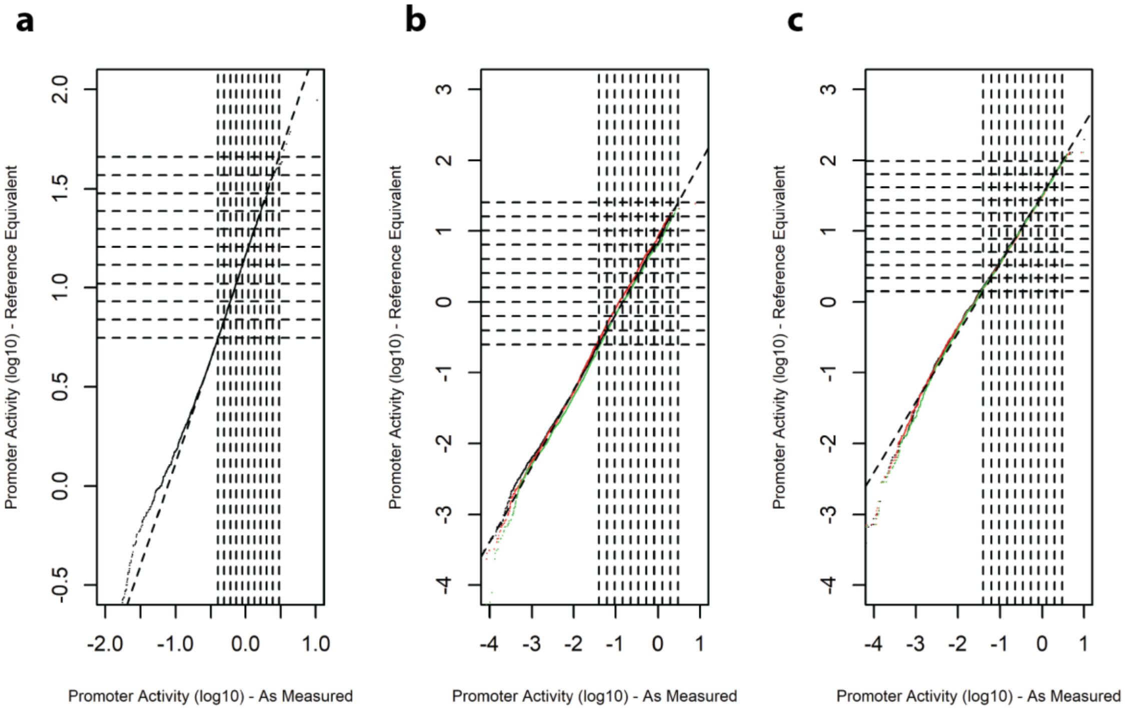

Supplementary Figure 25: FACS-seq bin edge initial calibration. Bin edges were rescaled in the P_{ZEV} experiment's Induced condition and in the validation experiment by collecting flow cytometry data for reference samples under reference conditions (those used for the P_{GPD} experiment) and those used for the final sorts. Linear models were fit between promoter activities measured in reference conditions (Reference Equivalent) and those used in the final experiments (As Measured). Bin edges set under as-measured conditions (vertical dashed lines) were then converted to corresponding values under reference equivalent conditions (horizontal dashed lines).

a: Calibration for P_{ZEV}-Induced condition.

b: Calibration for validation FACS-seq – uninduced condition.

c: Calibration for validation FACS-seq – induced condition.

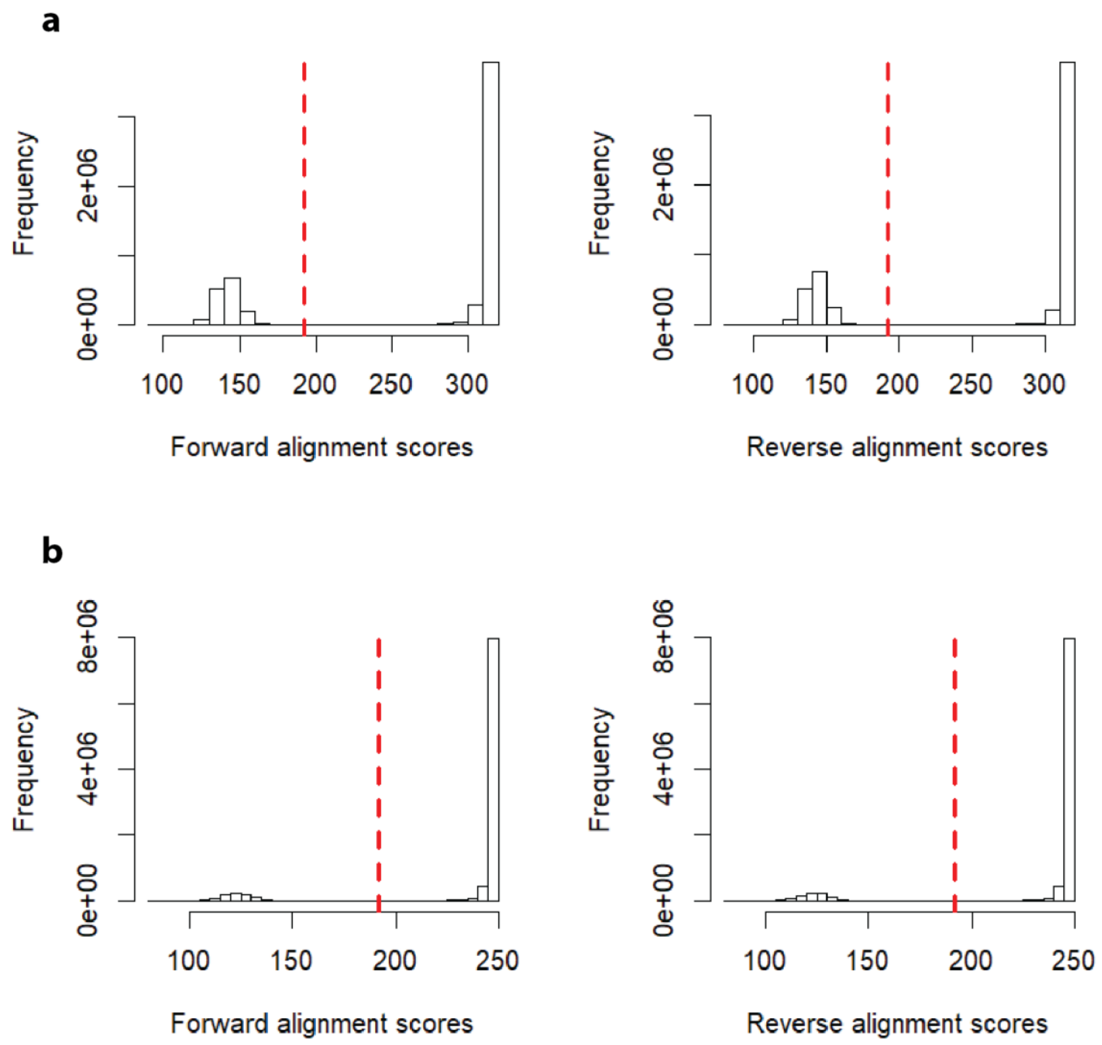

Supplementary Figure 26: Alignment scores in sorted reads during error-tolerant clustering in the P_{GPD} and P_{ZEV} experiments. Vertical dashed line indicates the threshold used to assign reads as either belonging to different clusters (left) or to the same cluster (right).

a: Clustering results in the P_{GPD} experiment.

b: Clustering results in the P_{ZEV} experiment.

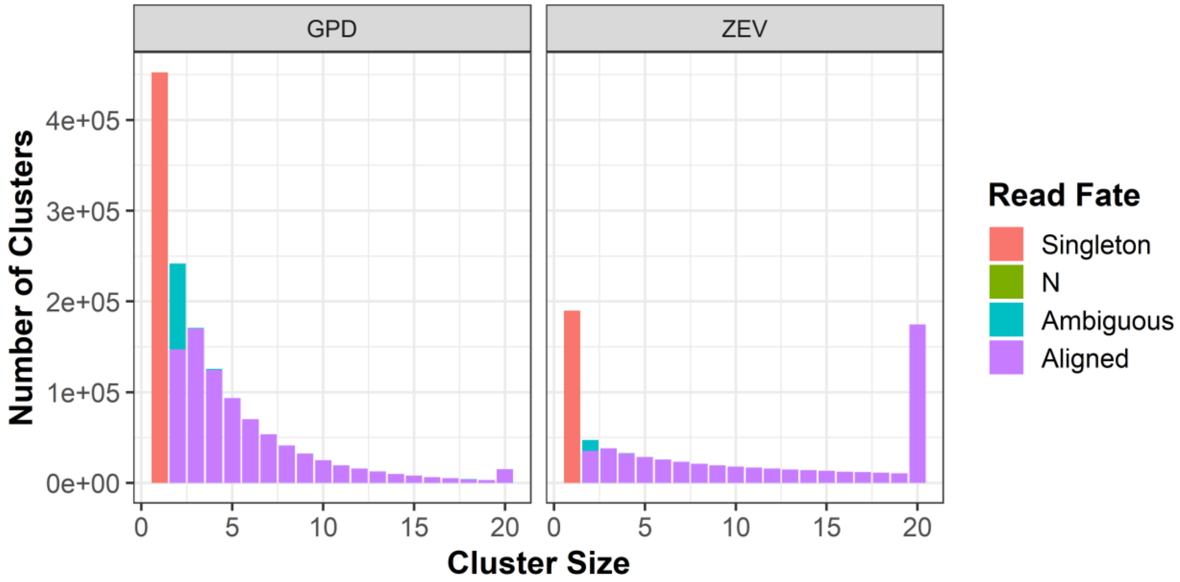

Supplementary Figure 27: Read cluster fates in consensus alignment, in the P_{GPD} and P_{ZEV} experiments. Read Fate: Clusters are either rejected as singletons, because one or more positions were uncalled in all reads (N), because one or more positions lacked a consensus as to the final base call (Ambiguous), or were successfully aligned. The bar for Cluster Size 20 contains all clusters with 20 or more reads in the MiSeq run.

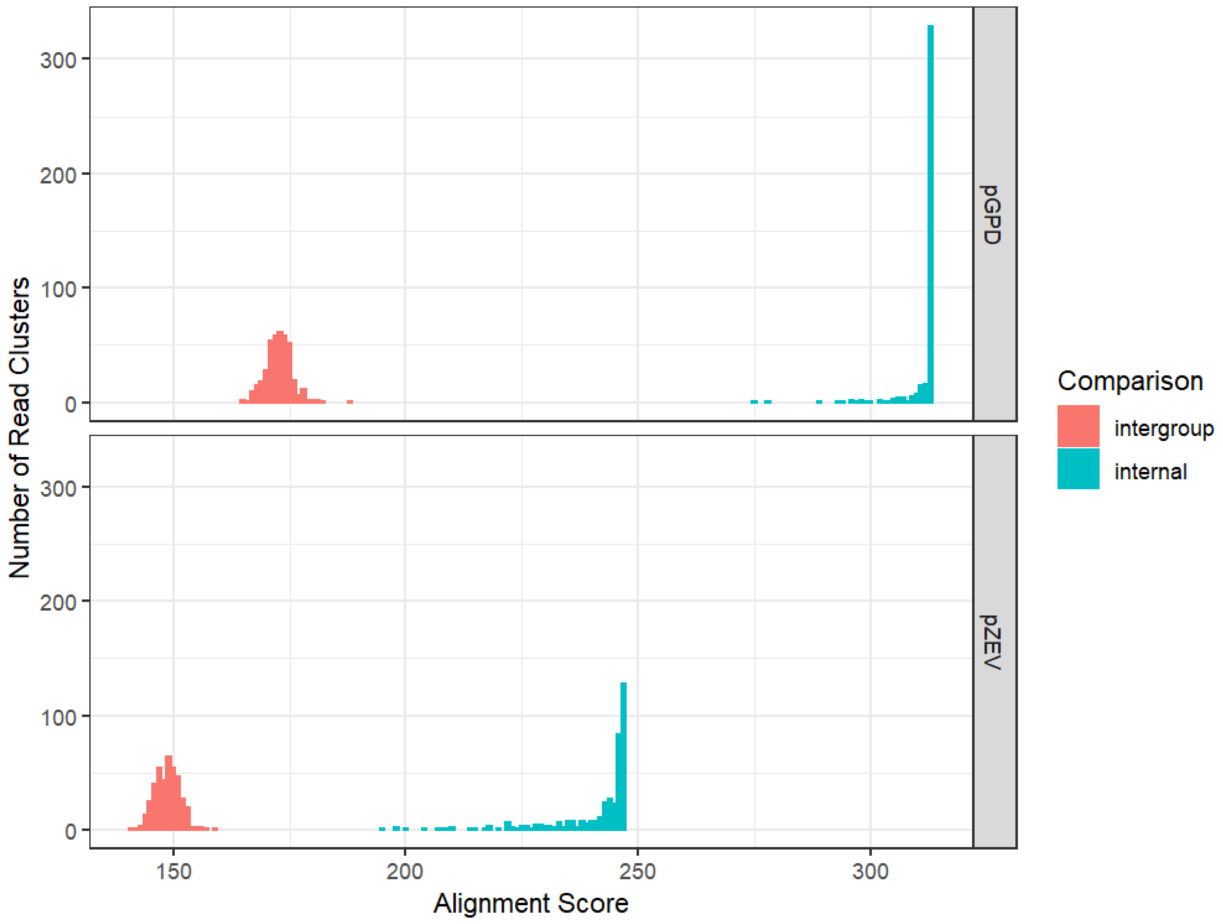

Supplementary Figure 28: Intergroup and internal alignment distances in consensus alignment, in the P$_{GPD}$ and P$_{ZEV}$ experiments. Data presented are for a random selection of 400 clusters in each experiment. Comparison: Approach used to generate alignment score. Intergroup: score presented is the highest (shortest distance) from the displayed read cluster's consensus sequence to that of any other sequence in the dataset. Internal: score presented is the lowest (longest distance) between any pair of sequences in the read cluster.

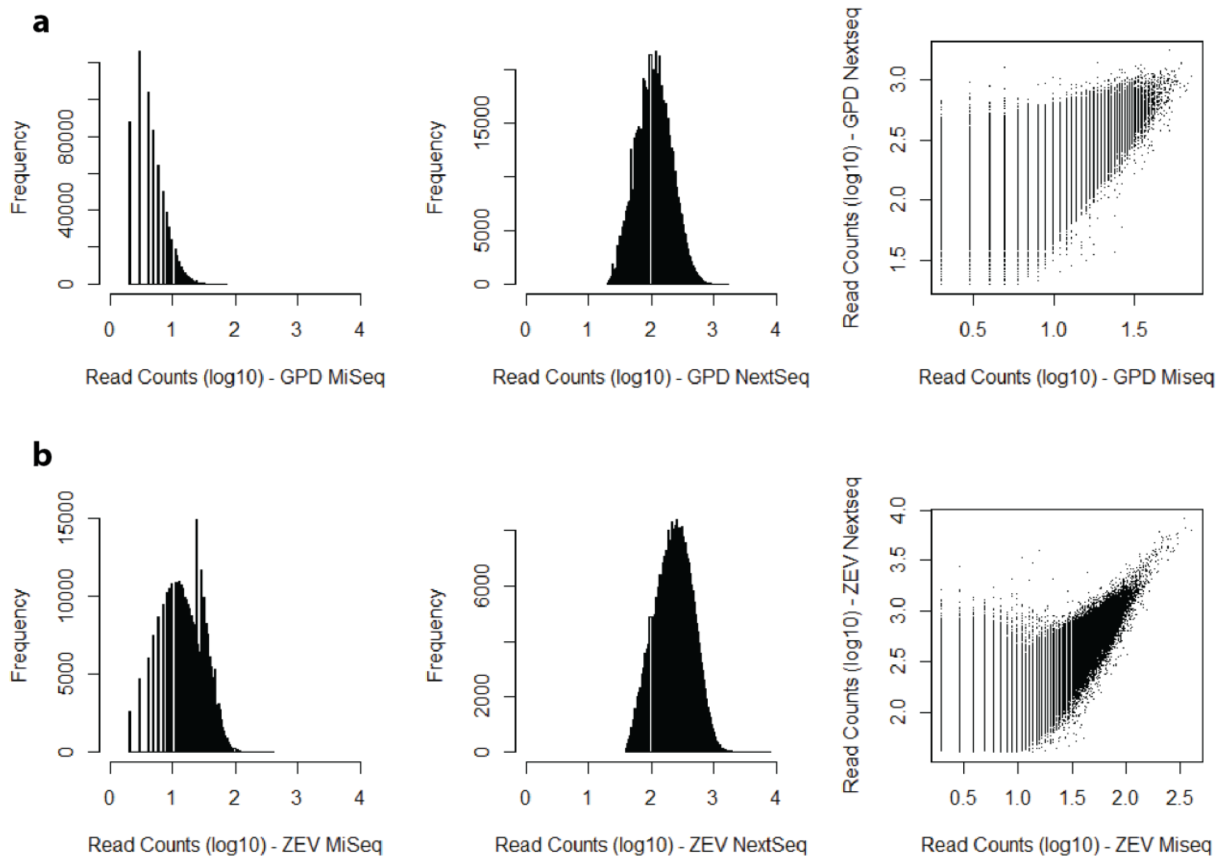

Supplementary Figure 29: Occurrence counts for each unique sequence in the MiSeq and NextSeq NGS runs for the P_{GPD} and P_{ZEV} experiments.

a: Read counts (on base-10 logarithmic scale) for each measured sequence in NGS runs for the P_{GPD} data: MiSeq (left), NextSeq (center), and NextSeq plotted directly against MiSeq (right).

b: As for **a**, but for NGS runs in the P_{ZEV} experiment.

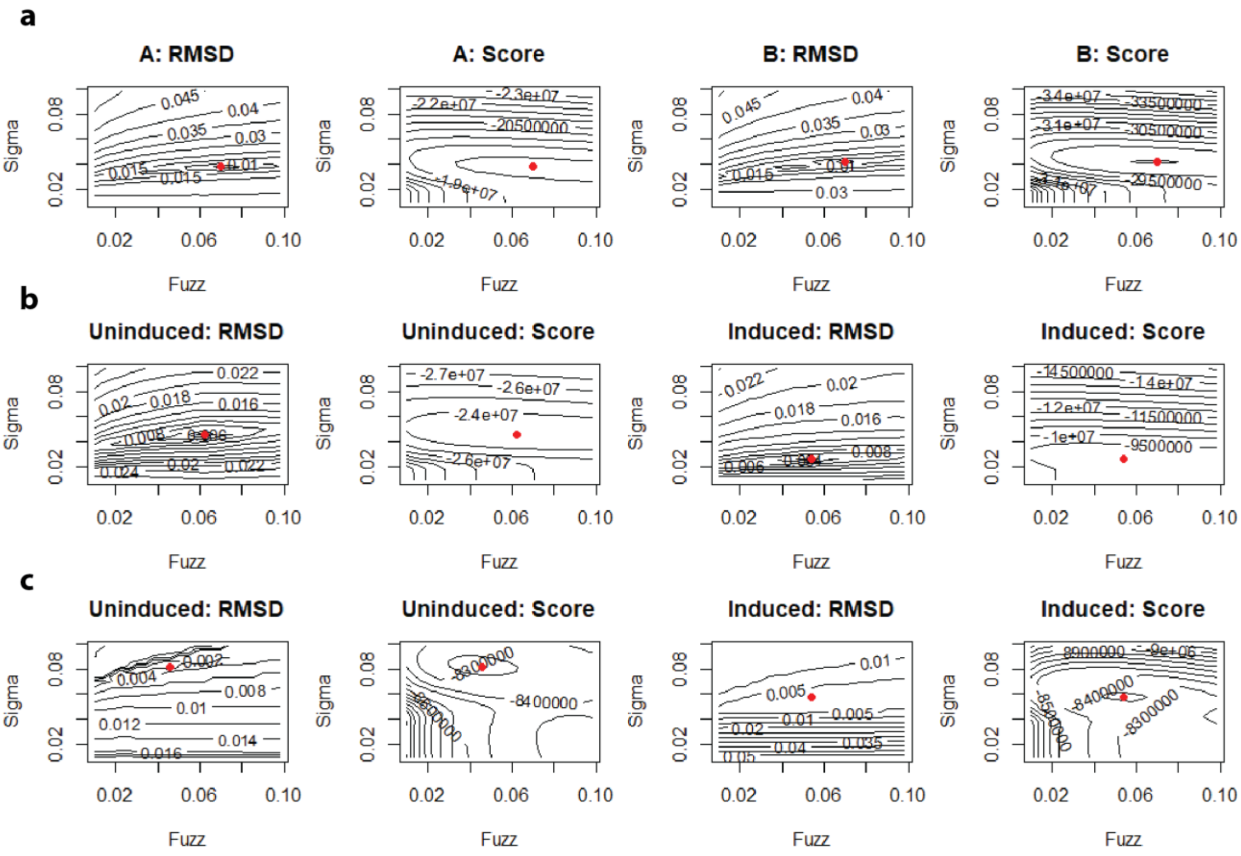

Supplementary Figure 30: Grid search for mean fitting hyperparameters in FACS-seq.

a: Hyperparameter search for the P_{GPD} experiment. A, B: experimental replicates. RMSD: root-mean-squared distance from the vector of fit means at a point to the final means. Score: log-likelihood maximized for parameter fitting. Sigma, Fuzz: hyperparameters to be optimized. Red dot: final hyperparameter values (given for all experiments in Supplementary Table 17).

b: Hyperparameter search for the P_{ZEV} experiment. Uninduced, Induced: Experimental conditions for this experiment (no beta-estradiol or 1 uM beta-estradiol added).

c: Hyperparameter search for the validation FACS-seq.

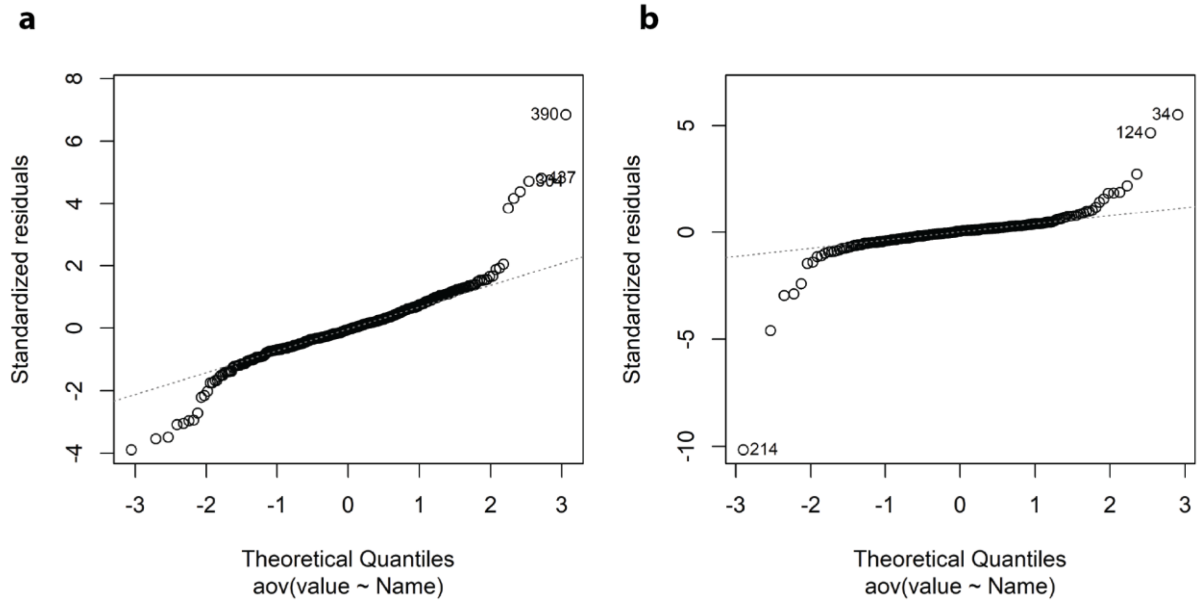

Supplementary Figure 31: Establishing normality of flow cytometry data with Q-Q plots.

a: Q-Q plot of residuals for promoter activity measurements in the uninduced condition. X-axis: theoretical quantiles for residuals of an ANOVA fit to single-sequence measurements of promoter activity (displayed in Figure 4), conditional on the sequences tested. y-axis: actual standardized residuals.

b: Q-Q plot of residuals for promoter activity measurements in the induced condition; axes as in **a**.

**Task**	**Single Dataset**	**Median Joined**	**Final**
P_{GPD}	0.65	0.72	0.80
P_{ZEV} – Uninduced	0.75	0.80	0.84
P_{ZEV} – Induced	0.73	0.75	0.79
P_{ZEV} – Activation Ratio	0.72	0.77	0.82

Supplementary Table 1: Coefficients of determination for model fits. The column Task describes the data being modeled; P_{GPD} is as in Fig. 2a, Supplementary Fig. 8a; P_{ZEV} – Uninduced is as in Fig. 2b, Supplementary Fig. 8b; P_{ZEV} – Induced is as in Fig. 2c, Supplementary Fig. 8c; and P_{ZEV} – Activation Ratio is an in Figs. Fig. 2d, Supplementary Fig. 8d. Column Single Dataset contains R^2 values for the single-dataset models (see Supplementary Fig. 8), column Median Joined contains the median R^2 values for the ensemble of nine submodels when carrying out each prediction task independently, and column Merged contains the R^2 values achieved by taking the average of submodel predictions as a final prediction.

ID	Pro-moter	Objective	GC Constr.	Extrap. Penalty	Design Strategy	Thresh.	Generated	Synth.	Measured (FACS-seq)	Measured (Indiv.)	Notes
18	GPD	Activity	-	-	Screening	0.45	113	113	89	3	-
19	GPD	Activity	-	+	Screening	0.45	112	112	21	2	-
20	GPD	Activity	-	-	Evolution	0.6	111	111	96	0	Threshold Reference
21	GPD	Activity	-	+	Evolution	0.6	113	113	87	4	In Silico
22	GPD	Activity	+	-	Evolution	0.6	110	110	1	5	-
23	GPD	Activity	+	+	Evolution	0.6	113	113	81	13	Final Promoter Set
24	GPD	Activity	-	-	Gradient	0.6	116	116	0	5	-
25	GPD	Activity	-	+	Gradient	0.6	112	112	45	3	-
26	GPD	Activity	+	-	Gradient	0.6	112	112	83	3	-
27	GPD	Activity	+	+	Gradient	0.6	111	111	96	4	-
28	GPD	Activity	-	+	Gradient	0.75	115	115	0	5	-
29	ZEV	Induced activity	-	-	Screening	1.45	115	115	80	0	-
30	ZEV	Induced activity	-	+	Screening	1.45	119	119	105	1	-
31	ZEV	Induced activity	-	-	Evolution	1.6	117	117	102	1	Threshold Reference
32	ZEV	Induced activity	-	+	Evolution	1.6	116	116	85	0	In Silico
33	ZEV	Induced activity	+	-	Evolution	1.6	113	113	102	1	-

34	ZEV	Induced activity	+	+	Evolution	1.6	112	112	99	14	Final Promoter Set
35	ZEV	Induced activity	-	-	Gradient	1.6	115	115	106	1	-
36	ZEV	Induced activity	-	+	Gradient	1.6	118	118	99	3	-
37	ZEV	Induced activity	+	-	Gradient	1.6	117	117	103	1	-
38	ZEV	Induced activity	+	+	Gradient	1.6	115	115	95	2	-
39	ZEV	Induced activity	-	+	Gradient	1.65	119	119	101	6	-
40	ZEV	Activation Ratio	-	-	Screening	1.75	115	115	82	3	-
41	ZEV	Activation Ratio	-	+	Screening	1.75	118	118	91	3	-
42	ZEV	Activation Ratio	-	-	Evolution	1.85	115	115	61	3	Threshold Reference
43	ZEV	Activation Ratio	-	+	Evolution	1.85	115	115	92	3	In Silico
44	ZEV	Activation Ratio	+	-	Evolution	1.85	110	110	89	11	Final Promoter Set
45	ZEV	Activation Ratio	+	+	Evolution	1.85	114	114	87	3	-
46	ZEV	Activation Ratio	-	-	Gradient	1.85	115	115	77	0	-
47	ZEV	Activation Ratio	-	+	Gradient	1.85	112	112	46	0	-
48	ZEV	Activation Ratio	+	-	Gradient	1.85	112	112	94	3	-

49	ZEV	Activation Ratio	+	+	Gradient	1.85	112	112	87	7	-
50	ZEV	Activation Ratio	-	+	Gradient	2	116	116	85	6	-

Supplementary Table 2: Design parameters and measurement outcomes for sets of designed promoters. GC Constr.: + if GC constraint applied to this design, - if not; Extrap. Penalty: extrapolation penalty; Thresh.: target predicted score for a sequence to be accepted for testing; Generated: sequences generated *in silico*; Synth.: sequences accepted for synthesis by Twist Bioscience; Measured (FACS-seq): sequences successfully measured in the validation FACS-seq experiment; Measured (Indiv.): sequences successfully measured in individual-sequence testing; Notes: Threshold Reference if used as a reference to set the threshold for designs with this objective, Final Promoter Set if chosen for additional characterization as a final design, In Silico if chosen for characterization by *in silico* mutagenesis, - otherwise.

ID	Promoter	Name	Description	Selected	Synth.	Measured (FACS-seq)	Measured (Indiv.)	Training Data
0	GPD	Top Outlier	Sequences only observed in Bin 12 in both replicates	20	20	20	0	+
1	GPD	Top No-Outlier	Non-outlier sequences with highest activity	40	34	28	0	+
2	GPD	Range	Sample across a range of GPD activities	80	79	0	5	-
3	GPD	Bottom Outlier	Sequences only observed in Bin 1 in both replicates	20	20	20	4	-
4	GPD	Test Outliers	Worst model predictions on GPD test data	80	80	2	5	-
5	GPD	Test Accurate Highly Active Predictions	GPD test sequences accurately predicted to be highly active	40	40	38	0	+
6	ZEV	Uninduced Bottom Outlier	Sequences only observed in Bin 1 in Uninduced condition	20	20	20	0	-
7	ZEV	Induced Bottom Outlier	Sequences only observed in Bin 1 in Induced condition	20	20	20	0	-
8	ZEV	Uninduced Top Outlier	Sequences only observed in Bin 12 in Uninduced condition	20	20	20	0	-
9	ZEV	Induced Top, No Outlier	Non-outlier sequences in Induced with highest activity	80	72	69	0	+
10	ZEV	High AR, No Outlier	Sequences with high activation ratios in ZEV data	40	28	25	2	+

11	ZEV	Grid	Grid sampling of uninduced and induced activities	216	210	189	4	-
12	ZEV	Uninduced Test Outliers	Worst model predictions on ZEV test data for Uninduced condition	80	41	41	2	-
13	ZEV	Induced Test Outliers	Worst model predictions on ZEV test data for Induced condition	80	70	66	1	-
14	ZEV	AR Test Outliers	Worst model predictions on ZEV test data for activation ratio	80	78	76	2	-
15	ZEV	Test Accurate Highly Active Predictions, Uninduced	ZEV test sequences accurately predicted to be highly active in Uninduced condition	40	40	37	0	-
16	ZEV	Test Accurate Highly Active Predictions, Induced	ZEV test sequences accurately predicted to be highly active in Induced condition	40	40	40	0	+
17	ZEV	Test Accurate Highly Active Predictions, AR	ZEV test sequences accurately predicted to have high activation ratios	40	40	39	3	+

Supplementary Table 3: Control promoter sets selected for the validation FACS-seq. Selected: sequences chosen for resynthesis; Synth.: sequences accepted for synthesis by Twist Bioscience; Measured (FACS-seq): sequences successfully measured in the validation FACS-seq experiment; Measured (Indiv.): sequences successfully measured in individual-sequence testing. Training Data: + if chosen as training data for comparison to designs, - if not.

**Name**	**Promoter**	**Objective**	**Promoter Set**	**Uninduced Activity**	**Induced Activity**	**Activation Ratio**
P_{GPD}-1	P_{GPD}	A	23	6.29 (5.31, 7.45)		
P_{GPD}-2	P_{GPD}	A	23	7.37 (5.84, 9.29)		
P_{GPD}-3	P_{GPD}	A	23	8.61 (6.58, 11.27)		
P_{GPD}-4	P_{GPD}	A	23	9.01 (6.73, 12.06)		
P_{GPD}-5	P_{GPD}	A	23	9.05 (7.77, 10.54)		
P_{GPD}-6	P_{GPD}	A	23	9.33 (7.03, 12.37)		
P_{GPD}-7	P_{GPD}	A	23	10.15 (8.74, 11.79)		
P_{GPD}-8	P_{GPD}	A	23	11.97 (11.15, 12.85)		
P_{GPD}-9	P_{GPD}	A	23	12.41 (10.01, 15.39)		
P_{GPD}-10	P_{GPD}	A	23	13.35 (12.06, 14.79)		
P_{GPD}-11	P_{GPD}	A	28	0.80 (0.60, 1.06)		
P_{GPD}-12	P_{GPD}	A	28	3.09 (2.66, 3.59)		
P_{GPD}-13	P_{GPD}	A	28	13.87 (11.44, 16.81)		
P_{GPD}-14	P_{GPD}	A	28	16.45 (12.88, 21.01)		
P_{GPD}-15	P_{GPD}	A	28	20.17 (17.12, 23.78)		

P_{GPD}-16	P_{GPD}	A	22	5.78 (5.21, 6.41)		
P_{GPD}-17	P_{GPD}	A	22	7.11 (7.03, 7.18)		
P_{GPD}-18	P_{GPD}	A	22	7.25 (6.94, 7.58)		
P_{GPD}-19	P_{GPD}	A	22	8.95 (7.75, 10.33)		
P_{GPD}-20	P_{GPD}	A	22	9.37 (8.10, 10.85)		
P_{GPD}-21	P_{GPD}	A	24	7.53 (3.58, 15.82)		
P_{GPD}-22	P_{GPD}	A	24	8.32 (6.82, 10.15)		
P_{GPD}-23	P_{GPD}	A	24	8.56 (8.02, 9.15)		
P_{GPD}-24	P_{GPD}	A	24	9.64 (9.02, 10.31)		
P_{GPD}-25	P_{GPD}	A	24	10.33 (8.64, 12.35)		
P_{ZEV}-I-1	P_{ZEV}	IA	34	0.79 (0.75, 0.85)	27.03 (24.76, 29.51)	34.02 (30.20, 37.96)
P_{ZEV}-I-2	P_{ZEV}	IA	34	0.56 (0.45, 0.69)	29.95 (26.50, 33.85)	53.83 (48.20, 59.58)
P_{ZEV}-I-3	P_{ZEV}	IA	34	0.29 (0.19, 0.45)	47.21 (42.21, 52.80)	162.80 (118.02, 218.91)
P_{ZEV}-I-4	P_{ZEV}	IA	34	0.32 (0.25, 0.41)	48.06 (45.85, 50.39)	149.58 (115.09, 190.40)

P_{ZEV}-I-5	P_{ZEV}	IA	34	0.40 (0.37, 0.43)	49.18 (46.14, 52.42)	123.42 (111.01, 136.05)
P_{ZEV}-I-6	P_{ZEV}	IA	34	0.33 (0.30, 0.37)	53.11 (48.55, 58.09)	161.29 (157.74, 164.63)
P_{ZEV}-I-7	P_{ZEV}	IA	34	1.27 (1.01, 1.61)	54.52 (40.60, 73.19)	42.79 (34.82, 51.73)
P_{ZEV}-I-8	P_{ZEV}	IA	34	0.66 (0.57, 0.77)	56.53 (46.02, 69.43)	85.37 (77.19, 93.67)
P_{ZEV}-I-9	P_{ZEV}	IA	34	0.44 (0.37, 0.52)	58.63 (51.12, 67.25)	133.63 (123.92, 143.25)
P_{ZEV}-I-10	P_{ZEV}	IA	34	0.65 (0.55, 0.77)	59.03 (48.69, 71.57)	90.74 (84.34, 97.06)
P_{ZEV}-I-11	P_{ZEV}	IA	34	0.96 (0.79, 1.18)	63.85 (53.30, 76.50)	66.41 (64.20, 68.51)
P_{ZEV}-AR-1	P_{ZEV}	AR	44	0.14 (0.13, 0.14)	12.28 (10.39, 14.52)	90.75 (76.37, 106.37)
P_{ZEV}-AR-2	P_{ZEV}	AR	44	0.06 (0.04, 0.08)	5.76 (4.98, 6.66)	98.28 (69.85, 134.59)
P_{ZEV}-AR-3	P_{ZEV}	AR	44	0.05 (0.04, 0.06)	5.88 (5.36, 6.45)	117.29 (101.92, 133.48)
P_{ZEV}-AR-4	P_{ZEV}	AR	44	0.05 (0.04, 0.06)	6.61 (6.06, 7.21)	133.25 (111.15, 157.46)
P_{ZEV}-AR-5	P_{ZEV}	AR	44	0.05 (0.04, 0.07)	8.86 (7.18, 10.93)	174.93 (110.92, 266.04)

P_{ZEV}-AR-6	P_{ZEV}	AR	44	0.05 (0.04, 0.06)	8.83 (2.12, 36.81)	194.91 (58.02, 594.51)
P_{ZEV}-AR-7	P_{ZEV}	AR	44	0.05 (0.04, 0.09)	10.88 (10.21, 11.60)	198.31 (123.04, 307.71)
P_{ZEV}-AR-8	P_{ZEV}	AR	44	0.05 (0.05, 0.06)	11.65 (11.02, 12.32)	220.27 (182.07, 262.46)
P_{ZEV}-AR-9	P_{ZEV}	AR	44	0.05 (0.04, 0.06)	12.35 (11.69, 13.04)	250.81 (219.48, 283.60)
P_{ZEV}-AR-10	P_{ZEV}	AR	44	0.05 (0.05, 0.05)	13.12 (11.72, 14.68)	258.73 (235.88, 281.71)
P_{CYC1}	C	C	C	0.19 (0.13, 0.26)		
P_{ADH1}	C	C	C	0.66 (0.63, 0.69)		
P_{TEF1}	C	C	C	1.52 (1.45, 1.59)		
P_{TPI1}	C	C	C	4.01 (3.81, 4.23)		
P_{PGK1}	C	C	C	6.14 (5.87, 6.42)		
P_{GPD}	C	C	C	14.65 (13.46, 15.94)		
P3	C	C	C	0.10 (0.07, 0.15)	56.47 (35.45, 89.95)	542.20 (348.14, 815.16)
P4	C	C	C	0.08 (0.03, 0.21)	8.20 (3.33, 20.24)	105.94 (54.57, 195.10)
P8	C	C	C	0.43 (0.40, 0.47)	5.43 (4.62, 6.37)	12.62 (9.97, 15.67)

Supplementary Table 4: Individually measured activities of final promoter designs and comparison promoters. C: Comparison promoter. Activities for final promoter designs are given in the absence of beta-estradiol for all; where measured, activities measured in the presence of 1 μM beta-estradiol, and calculated activation ratios, are also given. 95% confidence intervals are provided for all quantities measured.

Name	Sequence
P_{GPD}-1	TCCAGCCGGAAAAGCCTCGTTCAGAATGCCTTGGGTGTTATGTCTGGGTGTTCCATCACACGGCATCCAGGACGGCCG<br>TACGGTGCTGAGACACGGCATCCACATCCGATATCCCAGCCACGCGAAAGTGCCGTCCACTGTGTTGGCTGGTTCGCA<br>CGTTTGACACCGTCGAGATTTGGTGTTGTAGATCTCACGTATATAAAGCCGAGCTAAGTGTTCAACTTGATCTCGAGTTT<br>TACTAATCCTCGTCCTTTCGACTTCTTGAATCACACCAAGAAACCAAACTCAAACTCTCCCACTATAATACCCATT
P_{GPD}-2	CGGTGCCGTGTGCGGCGTTTGTGACTCAAGGAGTAGCATGTGTCTGGGTGTGAGAGCCCACGGCATCCATTTCATCGC<br>CGTTTCAGGCCGATACGGCATCCATTCTTTTTATCCCAGCCAGGTAATGCGCCGTTCGGCCAAAGCGCGTCGGCGTTC<br>GACGACTATTTTCAACGTCCCGACTAATCGGTGCCAACGTATATAAAGCGTCAAGTTTCGCTATTGTGGGAATCGAGTTA<br>CGATTATTCCCCGAACTGTTTTTTGATAGTTTCCACCAAGCCAACTATACTCTTAACTATCCACTACCAATCTACT
P_{GPD}-3	ACCTGCGCCTAACGTGGAAAATTTTCGCCGCACGTTTGGGTGTCTGGGTGGTTAAATAAACGGCATCCAATCATGCCAA<br>CGTCGAGCCCGGCACGGCATCCACTATTTCAATCCCAGCCAGGAATGGAACGGCACAAATTTCAGATTCTTCGGAGGC<br>CGAAATTGACAGGACACGTTGCCTTCCGATCCCACGAGTATATAAAGTCAGCGCTTTTTCGTCTTTCTACCAGAATGGTG<br>ATCTCGAGTTCCCATTCTTGTTAGAACGTTCTCACCAAGAAATCACAAACTTCTACTTACACCACTCTATAAATT
P_{GPD}-4	CGCTGCGGAAACCGACAATACGCAGGCTCATTATCACAGATGTCTGGGTGCACTACGGCACGGCATCCAATTTCCACTT<br>CCGTAAACGCGGCACGGCATCCAAATTATACATCCCAGCCATCTTGTTATCGGGGTCGGCTCTATTCTGTTTCACCGAG<br>TGTCGGAACCAATTAGCTCTGTTTACTCTTCGATTGGTATATAAAGGGATCAATCTAGTTGTGAAGTTATTCCTCATAAAT<br>CTCTCTCTCTCCTATTCTTCTCACATTTTTCACCAAGAACATCAACTCTTTCCCAACAAACTAATCCCCAATC
P_{GPD}-5	TATTTTTAGTCATATGGTGTGAACGGAATGAAAGACGAGGTGTCTGGGTGGAAACCCAGCTGGCATCCACTATACTAGT<br>GGAATTTTGAAGCCTGGCATCCAGTAATTACATCCCAGCCAGTGGGCACAGTTTGTTCCCTCTTTCCCGTGCCGCATGA<br>GTCGTAGTGCGATGGCTGGCAAAATTCTCAGGCGGTGTATATAAAGAAGCCATTCTGGAGCATATTTTTTCCTCTTGCTC<br>AGGATTTTGAACAATTAGTCTTCTTCTTTTGCACCAAGACTCTTATTAACTCTCCAACACTCTAACAATTAATC
P_{GPD}-6	TCTGTTCGGCATTTCTGGTCCGCCTTTTCCACGGCACTACTGTCTGGGTGGAGCCGCGCACGGCATCCATTTGCAAATT<br>CTTGTGGATGTAAACGGCATCCAATTAATGAATCCCAGCCAGCATACGAGTTAATTGCCAATCATCACGGTGCCGTGTG<br>AAGTATCAATCCCTGTTCCGCACGCCAGTACCAAACGTATATAAAGTCGCTCTTTTGGAACCACTCAGACAAACGAGTTC<br>CTATAACATTTCCTGGTTTATTCTTTCAAGACACCAAGACTCTCAATCTACAATTACTCACCTACTATCCTAAA
P_{GPD}-7	GAGGTGCCGCATGTGGGTAAAAAGGCAAGAAGTTTTAACGTGTCTGGGTGTTTGTCGCCGGGGCATCCAACACTAAAA<br>CGCCGCGTGACACACCGGCATCCAGTTTCAAAATCCCAGCCACGGCTCTGTTGAATTCGGATGAGTCACACTTTCTCGT<br>TTGATCGGGCCCACATTGCGTCCCATGTCAATTTGGCGTATATAAAGAGGCGAATATTCCAAGAGCACATCCAGTCACA<br>CTCTTCGGCTCGATTAACTTTGATGGTTGAGCCCACCAAGTCAACTAAATCCAACTCACATAACTTAACTATCACA
P_{GPD}-8	TCCTCGCGGGACTCATCTAGCCACAGATCGCCTCGACGGATGTCTGGGTGCGGCACCGTGCGGCATCCAAACGGCAC<br>GGGCACAATTCACACACGGCATCCACTTCCCCAATCCCAGCCATATTCGCGTTTTTTACGGCCTTCGGATGTCAACCAT<br>GATTTATTATTGGTGCATGTGAATCTCGACTTTGCGAAGTATATAAAGGGTGGCCTTCTTGAGCTCTCTCTTACCATATCC<br>TCTTGAGAAACTGATAATTTTCTTGTCATGATCACCAAGACATTACTCTCTTCCTAAATTCCACTCTCATACAAA
P_{GPD}-9	CAATTCGCAACGGTGCCGTGCACTCTTTTCGAGCCACCAATGTCTGGGTGTATTGCCGTACGGCATCCAAATGGTAGAA<br>CGACCTACGGCGTACGGCATCCACTCCTTGCATCCCAGCCACCGCTGGTTGCTTTCGGCCAACGCTGGATTACATTTCT<br>GCGATAATTAGCTTTTTCGCCACCTCCAGAAGAATCGTATATAAAGGGCGATCTTTTACTGTTTGTTTGACCTCAGGTGT<br>ATCCATTTGTTTTATTCGCAACATTTACCACCACCAAGAACAATATTCTAACTACTCACACCACTCTTACAAAA
P_{GPD}-10	TCGCCTTATTAGTCATGTCGACTTCGCCACTATAATCTTGTGTCTGGGTGTGCGAGCGTGCGGCATCCACGTGGCGACA<br>TATTTGAAAAGATACGGCATCCAGCCAGCCGATCCCAGCCATGACCTTGGACGCGGCACCAAACTCATTATGGCTTACA<br>CCTTTATCGCTTTCCTTTCGCCGTCCCAGCGGAAGCGTATATAAAGGGACGTAGATTTCATCTTTCAATGCCAGTCTTTT<br>CCTTTTCTGGTTTTTCCTCCATTAAAGAGAACACCAAGTCTCTTAAATATCCTACCCCCAATACTCTCTCTACT
P_{GPD}-11	CAGCCATTCGTGTGCACGCGCCGTGCGCACGCGCGAAAAATGTCTGGGTGCAATTTCGCACGGCATCCATTTCTCGCG<br>CGGCACCCCGTCGCACGGCATCCAATTTTTTCATCCCAGCCATGTGAATTTGAAAATTTCACATGCTGGTCACGACGTA<br>GCGGATGCAAATGTCCAACGCCTCGCCCGTCCGAAACGTATATAAAGAGCACATTTCTTCGACTGTTGATTCGTTCTCTT<br>GTTCTTCTCTCGATCACTATCTAGTACGAAATCACCAAGACTCCAAACTCTCTAAACTCATGATACCAACTCAAT
P_{GPD}-12	CAGTGCCGTGCGCACGGCACTTGCGCACGCGCACGGCACTTGTCTGGGTGCACTTTCGCACGGCATCCAGGTCACGC<br>ACACGCACCCGTCGCACGGCATCCAATTTTTTTATCCCAGCCATGTGAATTTCGACCTGCTCATTCTCATGTTTCGAGTG<br>ATGGCTGGTCAAACGACGTGTCCACCACTTTGGAAACGTATATAAAGAGCGACATTTTCTCGATCTCAACAAGTTTTCTC<br>TCCTCTATGATCTCTCTTACAAACGATCATATCACCAAGCTCAATAAATATCCTAAGATCACTAATGAACTCATG
P_{GPD}-13	CAGCAAATTTGTTGCCGTGTGCACGCGCTCGGCGCGAAAATGTCTGGGTGCACCCTCACACGGCATCCAATTCGCCGT<br>GCGTGACCCATCGCACGGCATCCAAAAATATTATCCCAGCCATGTGAATTTTCACATTCATTTATCTTGCTGGTTACTCG

	AAAAACCACGTCTCGTCTCACACGCGAAATTCAAACGTATATAAAGAGCGCATTTTGAATCGTTCTTGTTCGATCTTGTTC<br>TTCTCTAGTATCTCTAACGTAGATAAAGATCACCAAGAACTACAAACTCTCTAACTACTCTATAACATCCAAA
P_{GPD}-14	CAGCATTCGAAATTTTTCACATGGTGCCGTGCGGCGCGCCTGTCTGGGTGTATGGGTGTACGGCATCCAAGTGGCGCC<br>GTGTGCGCCCTCGCACGGCATCCAATTTTTTTATCCCAGCCATGTGAATTTTCTCATATGCACTTGCTTGGTTCACCCAC<br>CTGGTCATGAATTTTCACATGCACCTGTTCTGAAACGTATATAAAGAGCTCATATTTGATCTGATCTTCAACTTTTCGTCG<br>TTCTCTCTCTCTCTCAACTACGATCAAGATCACCAAGCTACAAACTAATATCCTACTACAATAACTACTTAGG
P_{GPD}-15	CAGCAAAATTTTTCACATGCGCGTCCGCGCATGTGAAAAATGTCTGGGTGCCGTGCGGCACGGCATCCAAAATTATGGT<br>CACGCGTCTCCGCACGGCATCCAAATTTTTCATCCCAGCCATCTCACTTTCACATTCTCATTCATGTGCTTCCAACATGT<br>TCGACGAATCGTTTCACGCGTCGTCGTCTGGAAACGTATATAAAGAGCGACAATTTGATCGTTCTATCTGAATTTCTCTC<br>TCGTTCTCTCTCTCTTACAATAATCTAAAACACCAAGCTCAATAAAACTATATAATATATCACTACAATCAAT
P_{GPD}-16	CGCGTGCCTTATCACATCGAGTCGCCGTGCATTGACAAATTGTCTGGGTGTAAGCCCTAGCGGCATCCAGTAAACGGA<br>CCAACATGGCTTGCTGGGCATCCAGTAATTACATCCCAGCCAATCCGCGTGACTCACGCGTTTTATTCCGGTTGAGCAT<br>GACGGTCATGCTTTTGAAGCCTCTGAAACGTCCAGGAGTATATAAAGGAGGTATCGGACTCGTAGCTTGCTCCCTCTCG<br>TTCGCCTGGCATCTTGTATTATGCTTACTCGCCCACCAAGCATCTAATAACATTTCCCATCACACACTATATAAAA
P_{GPD}-17	GCGGCACCAACTGCGTCGTGCAGTTTCAGCCTGCGGCTAATGTCTGGGTGTGCACCGGCACGGCATCCACTAACGGA<br>CAACCAAAATCCGGCTCGGCATCCACTAATCACATCCCAGCCAAATCGGTCAATTGGGAAATGGAAATTGTTGTGGTTG<br>GCAAAACTCGCAGATCACCAGATTGGCGAGGACGACAAGTATATAAAGGGGGGCGTTTTCTACTCTTCTCGATCTAGCT<br>TTTCGATTCTCGGCCCACTACTCATACTCCAGGCCACCAAGTTCTTCTTTTACCCCTTCAATAATCATACTCTAACT
P_{GPD}-18	CCTTCCAATTGCCGTGTCCTCTGAACGCGATTCGAGGGAATGTCTGGGTGTCGCCGATATCGGCATCCAGATTCACGG<br>ATTGCGATTAAGCCTCGGCATCCACTAATGCAATCCCAGCCACGGCACGGCATGATTAGAAAACTCCCTTGAGAGGACC<br>TGAGAAGTTCCCAAAACGGCGAACACTCACTGGTTAGGTATATAAAGCGTGTTCTTCTCAGTTTGTAGAAAGAATCTCTT<br>CTTCTTATAGACTTCCAATTTGGCGTTTCCAACACCAAGATCTTCTTATATCCACTATTCACTCATTACACAATC
P_{GPD}-19	TCATCATTGCTGAGTATCCGAGTTCACGGGGCCCGGAAGTTGTCTGGGTGCAGCCAGTTGCGGCATCCACTCTTTTCGT<br>CGTGGAAAGCCGCGCGGCATCCAAACTGTCAATCCCAGCCACGGCATTTAACGGCCCTCAAGCAGATGAAACGGCCAA<br>AATTCATCCGCCAAATCTCATCTCGTCGAGGGATTTTGTATATAAAGCCGAGTGTTCTTCTTCCTGGGACTTCCGGACTT<br>CTTCCTTTAACGATTCCCATTTCAGTAAAAAGCACCAAGTTCTACCTTTCATAACCCTATCCTATTAATCCTACT
P_{GPD}-20	CATTGGCTTGAAAAACAGATGCCGGAAACGGGAAGAAGAATGTCTGGGTGTCAACGGGTGCGGCATCCACTAAAGTAT<br>CGGGATTTACACGCCTGGCATCCACTTTTCCGATCCCAGCCAAGCATGACCAGTTCGGTCATGTATACGAAATTGCGAC<br>CCACACGAGGTGCAATCAGGGTGCTGCTGACCGAAAGGTATATAAAGGGCCTTCTCTTGCGTGGATAAGTCTCCTCAAT<br>TCTGATCTCAGATACGTTTTCCTTTCCGGTTCACACCAAGCCTCCAATACTCATTAACTAACCCTTAATCATACTA
P_{GPD}-21	TGGTAACGCTACCCACATGTGGTAAAACGTCGGTACGGGGTGTCTGGGTGTAAATAGATGCGGCATCCACTATTGGAC<br>GCTCGTCCCGTCGGCGGGCATCCACTAATTTCATCCCAGCCATCTGATTGATTCGAACGCGCCGTTCTCGCGACCGGC<br>AATCGCTCCAATCGGCCTGCTGCTTCGTGCGGTCAAACGTATATAAAGAGCGAACATTCGCTCTTATGAATGATTTCTCT<br>TTCTCTTCTTTTTGATTGGTTGTTATTCTAAATCACCAAGCCTCCACATATACACTCCAATATACCCCTACACACA
P_{GPD}-22	CAGCGCACGTGCGTCCGCTACCGCCAGCCCCACCGCGAGATGTCTGGGTGTTACGCGTTGCGGCATCCACTAAATAC<br>GCGCGCTAGCGCGAGCTGGCATCCACTAATTATATCCCAGCCATCTCGTGCTCGCCTGGAACCATACGAGTTCGCTAG<br>TGGTCGCCCGTCGTACGACGCTCGCTCATCTCGACAGACGTATATAAAGCGCGTATGTTTCGATACTTGTTCGATCTTG<br>TTCTTCTCTCTTCGATATATTTGTATTTTCTATAACACCAAGCACAATAAACATCTCTATCATCACAACTCAACCACT
P_{GPD}-23	CAGCTTCGCACGCACATGACGCGACGCCATACCCCGGAGATGTCTGGGTGTAAATGAGACTGGCATCCACTAACGCGC<br>GCGCGATCGGAACGCTGGCATCCACTAATTTCATCCCAGCCATCTGCTACACTTGTTCAGTGATACGAGCGAGCCAGA<br>GCGCGCGAGCGACCAGGAGCTCAGCGAGCGAACGAAACGTATATAAAGCGCTATAAATTTCGTTCGCTCGAGTTGCTC<br>TCTTGTCTCTCTCTCTCGTTCTTGATATATTATCTCACCAAGCACACATATATACAATCTCACCATCTCTATCACACA
P_{GPD}-24	CAGCAGCTCGACTCGCGCGGCGCGCGCGCTGCCCGTGAGATGTCTGGGTGTAGCATGATGCGGCATCCACTAACTGG<br>TCACGAGTAACGAATCGGGCATCCAGTAATATAATCCCAGCCATCTTTCAGACGTGCGCGCGCGTGGTCGCTGGTTGC<br>TAGCTCGTTTGGTCGATCGCGAGCGTGCTCTTTCGAGACGTATATAAAGAGCTCACATTCGATCCGTTCGTCTTAATTCT<br>CGTTGTTCGTCTTTCTTTTGTAATGATACTATATCACCAAGCTCAAAATCAAATCTATCACATATACATATCCCACA
P_{GPD}-25	CAGCGCACCCGCGTCTGCGCCATGAACCGGGGACCTGTGATGTCTGGGTGTAACTCCTTGCGGCATCCACTAAATACG<br>CACGCGCGCACAAGCGGGCATCCACTAATTTTATCCCAGCCATCTGAATGCCGAACCAGTCCGATTTGTCGGCACGCA<br>TGCGCGCGAGATGCCACGTCGCTGGTGCTCAAAAATTCGTATATAAAGAGCAACGAATTTTTCGCTACGTTTTTCTTTCT<br>CTGATGATCTCTCTTTTGATATTTTGTTCAAATCACCAAGCCCCATACATCTCTCATATACTCACTATCTCCCACA
P_{ZEV}-I-1	CTTTATATATATGCCACACCTGGTTTCGACGCAGTCGGCTGCGTGGGCGAATGGGAGCGTGGGCGTATAAACACGCGT<br>GGGCGGAGTCACTGTGGGGGGTCATACAGGTCAACGGCAGGAATAAGTATATAAAGACGGAAGGAAGCACTGCTCCCT

	CGATCCCGCCCTTCCTACTTTCTATAGCTTCTGTTCCTCGAAAGCAACACCAAGATTTCATAACTCAAACTCACACATACT<br>CTTTTAATC
P_{ZEV}-I-2	CTTTATATATAAGAGATACGCTAAACCGGCGCAGGGCGTTGCGTGGGCGTAGTGGAGCGTGGGCGGAAGAAAGTGCG<br>TGGGCGGTACGATCCAAATTCTATCCACTGAGTGCAGTCTCGGATAAGTATATAAAGACGGGAAGTTTAACTAGATACA<br>CCACGACCGGGCTCCCTTAGGTCTTTTTCGTTCTCAATTCTCAGCTTCACCAAGAACTCAAACCTACTTCCCACAACTCA<br>ATTACCAAAC
P_{ZEV}-I-3	AGTTTTGAGCTGCCCTTTATATATACTCGTTCTGGAGGTAGCGTGGGCGTGCCAATGCGTGGGCGGTCAGCATTGCGT<br>GGGCGGAAACCACGTTATAGAGGCAGCCTAAATTTCTGGCAGATAAGTATATAAAGACGGGTATGGTCAGTGGCCTACT<br>TCTCCCGATTATACTCGTCGCTTCACTAGCTCGTTATTTTTGCATACACCAAGACTCCAAAACTTTCCTCATACTTACAAC<br>TACATATC
P_{ZEV}-I-4	AAGGAGCTGCCTTTATATATATACTGGAGCTGTTGAACATGCGTGGGCGGGCAAACGCGTGGGCGTCCAAACGTGCGT<br>GGGCGTCTTCCGATTAGGGCTGGAGGCATTGGCCTCTTGGGAATAAGTATATAAAGACGGATGACTTACGGAGAACTC<br>ATATGATCTCGTTATTCCATACTTGCATGGTTTTCGATAACTCCGTTCACCAAGTCAACTCCAAACTATCTCCATACATAC<br>ATAAACAAA
P_{ZEV}-I-5	CTTATATATATGTTGGTGGTCCAGCTGATCCTCGTGTTTGGCGTGGGCGTGTTTCCGCGTGGGCGTGAGTCTATGCGTG<br>GGCGTGAGGGGGGTCAGTACAAGGATATGGCTAACTGGCGAATAAGTATATAAAGACGGCTCCCAAAAGGTTGATCGT<br>GGTGTCTCCTTCCCGGTCTTAAACTTGCTTCGTGCTAGTCTTACCACACCAAGTCCCACAAACTCTACTCACACCCCAAA<br>ATCCTAATC
P_{ZEV}-I-6	CTTTATATATAGATTGGACACCCCCCGGGACCATGAAACTGCGTGGGCGACGAAGTGCGTGGGCGTATCGCTGTGCGT<br>GGGCGGGTTCTGCTTTGCGTGTGATAACTAGTGATGCGATGGATAAGTATATAAAGACGGCAACTCTCATCAGTTCAGC<br>CTGAGAGTTTGAGTACTATCTAGACTGTCTAACTTGAGTTTCTAGACACCAAGCACATACTACTCTCCCCAATTCCCTATA<br>AACACAAA
P_{ZEV}-I-7	CTTTTATATAGTCAAGATTGCTCACAGCGGCCACATTCATGCGTGGGCGTTTCTTCGCGTGGGCGTTGATGAGTGCGTG<br>GGCGCGATGATCAGTAGCCCTACGTGACGCCCCCGGCCGAAATAAGTATATAAAGACGGGTTAATCTTCCTCTTGTAAG<br>ACAAAGTTCCCTCTTACTTCTGATAGCATAATACAATCTTCCACTCACCAAGATAACAAAATACACTCAAACACACTTCCT<br>ATATAAA
P_{ZEV}-I-8	ACCTTTATATATATGTCCTCAGGATGGAAAGAGATCTGACGCGTGGGCGGAGAATTGCGTGGGCGCGTGGAAACGCGT<br>GGGCGGTGTAGCGAACCCGCTATCCTTTCTAGTGACAGCCCAATAAGTATATAAAGACGGCCTTCTTGCAAATATGAGG<br>TCGTCTAAGTTTATACGCTAATTCTTCCATTCCTTAGTTTGAATCACACCAAGTTTCTAAATACACTTTCCTCATCTCTATT<br>ATCTATC
P_{ZEV}-I-9	CTTTTATATATCTGGGATAGTTCCTTTAGCCGGCGTGTTAGCGTGGGCGTTCATGCGCGTGGGCGAGGAGTATTGCGTG<br>GGCGTTAAGTGCTTATCCGCTGCGTCGCTGTGGCTCGTGCAATAAGTATATAAAGACGGATCTTACTGTAGAATTTGTAT<br>CATTGACTGTTTGTTAGTTACTTGTTATCCCTTGAATAGAAGCCCACCAAGAATCTATAACTCTTTACTCTTCATATTCCTT<br>CTATC
P_{ZEV}-I-10	CTTTATATATGTGAAGTGTTCGTGAAAGAAAAGCCCAGATGCGTGGGCGTTGTACAGCGTGGGCGTGTCGGAACGCGT<br>GGGCGTTCGATAACTCACGTTCTTGGCGTCGATCACTTCCCCATAAGTATATAAAGACGGCGAGGTGATGGGGATTCTG<br>TTTGAACCATGCTCGGTCGTTGTGTTGTTGTTCGATTCACTTAAAACACCAAGTCTCAATAAAACAAACTCCTATCAAACA<br>TCCCCATA
P_{ZEV}-I-11	TGCTGTATTTATATACTGGTGGAAGTGGTAAAATGTTGATGCGTGGGCGTGACGCTGCGTGGGCGTTCTGCGTTGCGT<br>GGGCGTTTACGCAGGAAATGATGGAGCCGTGTATGTCGCCGGATAAGTATATAAAGACGGTGGATTAGCCATAACTATT<br>TGTCGTAGGTTGTAGGAATTAACCTTTGTCTTTGGGATTTTGGATGCACCAAGACTAAAAACACTAACAAACTTCATAAAC<br>TCTCAATC
P_{ZEV}-AR-1	ACTTTTATATAGCGGTAATGATTTGATTGAACTTGGCATGGCGTGGGCGTACCCTTGCGTGGGCGTCTATAAAGGCGTG<br>GGCGGCTATGAAAAGGGGTATGCTTCCGCCTCTTGGAGCCAATAAGTATATAAAGACGGTGACAAATAAGGATCACTAG<br>TTCACAGGCGATCCCTGCTTCCGCTGGGTGGCTATCTTTTTTGAACACCAAGACTCACCCAACTCACTACTTCTCCCCAC<br>TCCCAACT
P_{ZEV}-AR-2	CGGCGAACGAAACTACATTCGGTGAAACTATCGACCGTACGCGTGGGCGTTGCAGAGCGTGGGCGGCTATAGTGGCG<br>TGGGCGGCTATCCCATGGGCGCTTGCCGCCCTTAATCGTGGACATAAGTATATAAAGACGGTGGAAGGCCGACCTACA<br>AGCGAGATAATCCCTTTCCTCTGGAGATGGTGTTCTAAGGGTCAGGCTCACCAAGCTACTCAAACCACCCCCTTCCAAT<br>CCTCTCATTATC
P_{ZEV}-AR-3	GGTCCTTCCACCCAGATACGGTGACTGTGGAACTTTGCGAGCGTGGGCGCTTATCTGCGTGGGCGGCTATTGCAGCGT<br>GGGCGGCTAAACCTCGAGAGGTTTGGGAGACTCCTACGATCGATAAGTATATAAAGACGGATCTGTGCTCACCCTCAAC

	TAATAGGGATTACCTTGCGATCCATTGGGTGCTCTGGATCGCAATGCACCAAGCATTTCTCCTTTTCAACTCCTCAACAATAACTCATT
P_{ZEV}-AR-4	ACGTCGGTTTGGTTAACACATTGATAGGGACACCAACCTTGCGTGGGCGGTTAAACGCGTGGGCGCTTATGATCGCGTGGGCGGCTATGTGGAGGAGATAGAATGGTTACCATGGGGACAATAAGTATATAAAGACGGATAAATGTTCTGTATAACCAAAGTCCTTCTCGATGTTTGTGAGTTTAGGCAGAAAGTATCAGATCCACCAAGCCAATTTTACACCTCTCCCACTACATTCATATTAAT
P_{ZEV}-AR-5	GGCTCAAAGTGGGCCTGTTAGTGGCATATTATCCTGGTGGGCGTGGGCGCTATATCGCGTGGGCGGCTAATTGTGCGTGGGCGGCTAGTTGCTTTTCATAACCGATTACCAAGGGCTCCTATAAGTATATAAAGACGGTGTGCTGTTGCCAGACTCTTGAGACACTGCAGGAATTCGCAATCTTCAGTTCTTACACGATAGCCCACCAAGCCCCCTTTAACTACCCCACTTCCATTTTTTCTAACC
P_{ZEV}-AR-6	AGCTTATATATAAGTCCTACAGTTAGTTACCTTCTATGGAGCGTGGGCGTAAGAGAGCGTGGGCGTCGTCGACAGCGTGGGCGTCAAAGGCGGCTACGGTTGGCCTTAATCTCCTCTCCAATAAGTATATAAAGACGGATAATCATCTAGAGTACACTTAGAGTGGGTCTATTGAATAGCCATTTTGGCGTCGTAGTCAGGAACACCAAGACCTCACCCTATTTCTCATTACTTAATCCCCTATCC
P_{ZEV}-AR-7	TGTTTATATAAGGTCGCGTGCCCGTTAGCGGTACTGAATAGCGTGGGCGTGGTCCTGCGTGGGCGGCTGTACCTGCGTGGGCGGCTATACTAACATAAGGCTCTTAGTAGTGAGTGTTGCATAAGTATATAAAGACGGAAGATTGAGAGACTAGAATTACCATTCCTGCATGGGTAAGATCCTCGTCCTTACCTAGCGTAACTCACCAAGTACAATATTTTCCACTCCCAAATCCTCATCATTCCA
P_{ZEV}-AR-8	CTAGGACGTTTGGTTGGGATCAACGCGGGAAGCGATCCATGCGTGGGCGGCTAAAGGCGTGGGCGGCTAGGTGAGCGTGGGCGAAGTGAAGGGCGATGGGCAAGAGACTCTTCACGATCCATAAGTATATAAAGACGGGCATATGTCCAACTGTACGCAGACGAGAACTGGATTGAACTTTGATCTCTGTACTCTTAGATTAGCACCAAGTTCAAACATACCCCTTCCTTCTAAACCTATTACATC
P_{ZEV}-AR-9	TGAGACGATACCATGGCGGGTACCTATAATGCGGTTCTGTGCGTGGGCGCACTCGAGCGTGGGCGGCTAATTATGCGTGGGCGGCTGCTGCGGTAGTCATAGCTTGAGGTATGGTGCTCAATAAGTATATAAAGACGGTATCTTCCTATCGTTTGGCTATCATAGTGGAGAAGTGCACCTCTCTATCACACTTGTGGCAATTTCACCAAGAACACAATTACTCTACATCCCCTCACTAAATTAAAT
P_{ZEV}-AR-10	TTGACTGTTTTATATAACCGACGGATCTTACAACCAGTGTGCGTGGGCGCCTAAAGGCGTGGGCGTGATGACGAGCGTGGGCGGCTAAGTGTCTGAAGTTGCTACTCCAAGGTGAGTTGGATAAGTATATAAAGACGGGTCTTTCTGTCCTCGCAGCTCAGGTACATGATGGTTTCTTATAGATCTTAGTTGAAGCTGAGATACACCAAGCTACACTTTATTCTTTCTACTAACCCTCCCTCTATT
P_{CYC1}	GAGCGTTGGTTGGTGGATCAAGCCCACGCGTAGGCAATCCTCGAGCAGATCCGCCAGGCGTGTATATATAGCGTGGATGGCCAGGCAACTTTAGTGCTGACACATACAGGCATATATATATGTGTGCGACGACACATGATCATATGGCATGCATGTGCTCTGTATGTATATAAAACTCTTGTTTTCTTCTTTTCTCTAAATATTCTTTCCTTATACATTAGGACCTTTGCAGCATAAATTACTATACTTCTATAGACACACAAACACAAATACACACACTAAATTAATATATACA
P_{ADH1}	GCATGCAACTTCTTTTCTTTTTTTTTCTTTTCTCTCTCCCCCGTTGTTGTCTCACCATATCCGCAATGACAAAAAAATGATGGAAGACACTAAAGGAAAAAATTAACGACAAAGACAGCACCAACAGATGTCGTTGTTCCAGAGCTGATGAGGGGTATCTCGAAGCACACGAAACTTTTTCCTTCCTTCATTCACGCACACTACTCTCTAATGAGCAACGGTATACGGCCTTCCTTCCAGTTACTTGAATTTGAAATAAAAAAAAGTTTGCTGTCTTGCTATCAAGTATAAATAGACCTGCAATTATTAATCTTTTGTTTCCTCGTCATTGTTCTCGTTCCCTTTCTTCCTTGTTTCTTTTTCTGCACAATATTTCAAGCTATACCAAGCATACAATTATACA
P_{TEF1}	ATAGCTTCAAAATGTTTCTACTCCTTTTTTACTCTTCCAGATTTTCTCGGACTCCGCGCATCGCCGTACCACTTCAAAACACCCAAGCACAGCATACTAAATTTCCCCTCTTTCTTCCTCTAGGGTGTCGTTAATTACCCGTACTAAAGGTTTGGAAAAGAAAAAAGAGACCGCCTCGTTTCTTTTTCTTCGTCGAAAAAGGCAATAAAAATTTTTATCACGTTTCTTTTTCTTGAAAATTTTTTTTTTTGATTTTTTTCTCTTTCGATGACCTCCCATTGATATTTAAGTTAATAAACGGTCTTCAATTTCTCAAGTTTCAGTTTCATTTTTCTTGTTCTATTACAACTTTTTTTACTTCTTGCTCATTAGAAAGAAAGCATAGCAATCTAATCTAAGTTTTGCCGCGGGAAATA
P_{TPI1}	CGGACCTTAATACATTCAGACACTTCTGCGGTATCACCCTACTTATTCCCTTCGAGATTATATCTAGGAACCCATCAGGTTGGTGGAAGATTACCCGTTCTAAGACTTTTCAGCTTCCTCTATTGATGTTACACCTGGACACCCCTTTTCTGGCATCCAGTTTTTAATCTTCAGTGGCATGTGAGATTCTCCGAAATTAATTAAAGCAATCACACAATTCTCTCGGATACCACCTCGGTTGAAACTGACAGGTGGTTTGTTACGCATGCTAATGCAAAGGAGCCTATATACCTTTGGCTCGGCTGCTGTAACAGGGAATATAAAGGGCAGCATAATTTAGGAGTTTAGTGAACTTGCAACATTTACTATTTTCCCTTCTTACGTAAATATTTTTCTTTTTAATTCTAAATCAATCTTTTTCAATTTTTTGTTTGTATTCTTTTCTTGCTTAAATCTATAACTACAAAAAACACATACATAAACTAAAATATACA
P_{PGK1}	AGGCATTTGCAAGAATTACTCGTGAGTAAGGAAAGAGTGAGGAACTATCGCATACCTGCATTTAAAGATGCCGATTTGGGCGCGAATCCTTTATTTTGGCTTCACCCTCATACTATTATCAGGGCCAGAAAAAGGAAGTGTTTCCCTCCTTCTTGAATT

	GATGTTACCCTCATAAAGCACGTGGCCTCTTATCGAGAAAGAAATTACCGTCGCTCGTGATTTGTTTGCAAAAAGAACAA<br>AACTGAAAAAACCCAGACACGCTCGACTTCCTGTCTTCCTATTGATTGCAGCTTCCAATTTCGTCACACAACAAGGTCCT<br>AGCGACGGCTCACAGGTTTTGTAACAAGCAATCGAAGGTTCTGGAATGGCGGGAAAGGGTTTAGTACCACATGCTATGA<br>TGCCCACTGTGATCTCCAGAGCAAAGTTCGTTCGATCGTACTGTTACTCTCTCTCTTTCAAACAGAATTGTCCGAATCGT<br>GTGACAACAACAGCCTGTTCTCACACACTCTTTTCTTCTAACCAAGGGGGTGGTTTAGTTTAGTAGAACCTCGTGAAACT<br>TACATTTACATATATATAAACTTGCATAAATTGGTCAATGCAAGAAATACATATTTGGTCTTTTCTAATTCGTAGTTTTTCAA<br>GTTCTTAGATGCTTTCTTTTTCTCTTTTTTACAGATCATCAAGGAAGTAATTATCTACTTTTTACAACAAATATATACA
P_{GPD}	GAGTTTATCATTATCAATACTCGCCATTTCAAAGAATACGTAAATAATTAATAGTAGTGATTTTCCTAACTTTATTTAGTCA<br>AAGAATTAGCCTTTTAATTCTGCTGTAACCCGTACATGCCCAAAATAGGGGGCGGGTTACACAGAATATATAACATCGTA<br>GGTGTCTGGGTGAACAGTTTATTCCTGGCATCCACTAAATATAATGGAGCCCGCTTTTTAAGCTGGCATCCAGAAAAAAA<br>AAGAATCCCAGCACCAAAATATTGTTTTCTTCACCAACCATCAGTTCATAGGTCCATTCTCTTAGCGCAACTACAGAGAA<br>CAGGGGCACAAACAGGCAAAAAACGGGCACAACCTCAATGGAGTGATGCAACCTGCCTGGAGTAAATGATGACACAAG<br>GCAATTGACCCACGCATGTATCTATCTCATTTTCTTACACCTTCTATTACCTTCTGCTCTCTCTGATTTGGAAAAAGCTGA<br>AAAAAAAGGTTGAAACCAGTTCCCTGAAATTATTCCCCTACTTGACTAATAAGTATATAAAGACGGTAGGTATTGATTGTA<br>ATTCTGTAAATCTATTTCTTAAACTTCTTAAATTCTACTTTTATAGTTAGTCTTTTTTTTAGTTTTAAAACACCAAGAACTTAG<br>TTTCGAATAAACACACATAAACAAACAAA
P3	TTATATTGAATTTTCAAAAATTCTTACTTTTTTTTTGGATGGACGCAAAGAAGTTTAATAATCATATTACATGGCATTACCA<br>CCATATACATATCCATATCTAATCTTACTTATATGTTGTGGAAATGTAAAGAGCCCCATTATCTTAGCCTAAAAAAACCTG<br>CGTGGGCGTCTCTTTGGAACTTTCAGTAATACGCTTGCGTGGGCGAACTGCTCATTGCTATATTGAAGTCCGTGCGTCC<br>TCGTCTTCACCGGTCGCGTTCCTGAAACGCAGATGTGCCTAACAATAAAGATTCTAGCGTGGGCGCAATACTAGCTTTT<br>ATGGTTATGAGCGTGGGCGAGAGGAAAAATTGGCAGTAACCGCGTGGGCGTGGCCCCACAAACCTTCAAATTAACGAA<br>TCAAATTAACGCGTGGGCGAACCATAGGATGATAATGCGATTAGTTTTTTAGCCTTATTTCTGGGGTAATTAATCAGCGA<br>AGCGATGATTTTTGATCTATTAACAGATATATAAATGGAAAAGCTGCATAACCACTTTAACTAATACTTTCAACATTTTCAG<br>TTTGTATTACTTCTTATTCAAATGTCATAAAAGTATCAACAAAAAATTGTTAATATACCTCTATACTTTAACGTCAAGGAGA<br>AAAAACTATATTTGCCGCCCAAAGAGCCGAACGAACTACCTTAACA
P4	TTATATTGAATTTTCAAAAATTCTTACTTTTTTTTTGGATGGACGCAAAGAAGTTTAATAATCATATTACATGGCATTACCA<br>CCATATACATATCCATATCTAATCTTACTTATATGTTGTGGAAATGTAAAGAGCCCCATTATCTTAGCCTAAAAAAACCTTC<br>TCTTTGGAACTTTCAGTAATACGCTTAACTGCTCATTGCTATATTGAAGTCCGTGCGTCCTCGTCTTCACCGGTCGCGTT<br>CCTGAAACGCAGATGTGCCTAACAATAAAGATTCTACAATACTAGCTTTTATGGTTATGAAGAGGAAAAATTGGCAGTAA<br>CCTGGCCCCACAAACCTTCAAATTAACGAATCAAATTAAGCGGCCGCGTGGGCGTTACTCAAGGCGTGGGCGTGCGTG<br>GGCGGGCGTGGGCGTGCGTGGGCGTCTAGACAACCATAGGATGATAATGCGATTAGTTTTTTAGCCTTATTTCTGGGG<br>TAATTAATCAGCGAAGCGATGATTTTTGATCTATTAACAGATATATAAATGGAAAAGCTGCATAACCACTCTAACTACTAC<br>TGTCAACATTCTCAGTGTGTATTGCTTCTTATTCAAATGTCATACAAGTATCAACAACAAATTGTTAATATACCTCTATACT<br>GTAACGTCAAGGAGAAAAAACTATATTTGCCGCCCAAAGAGCCGAACGAACTACCTTAACA
P8	GCGTGGGCGAATTGGTGCGTGGGCGCCAATTGGTGCGTGGGCGTCGAGCAGATCCGCCAGGCGTGTATATATAGCGT<br>GGATGGCCAGGCAACTTTAGTGCTGACACATACAGGCATATATATATGTGTGCGACGACACATGATCATATGGCATGCA<br>TGTGCTCTGTATGTATATAAAACTCTTGTTTTCTTCTTTTCTCTAAATATTCTTTCCTTATACATTAGGACCTTTGCAGCATA<br>AATTACTATACTTCTATAGACACACAAACACAAATACACACACTAAATTAATATTTGCCGCCCAAAGAGCCGAACGAACTA<br>CCTTAACA

Supplementary Table 5. Sequences of final promoter designs and comparison promoters.

Plasmid	Description	Ref.
pCS1748	Centromeric *URA3*; P_{TEF1}-GFP-T_{ADH1}; P_{TEF1}-mCherry-T_{CYC1}	1
pCS2656	*attL1*-P_{GPD}-*T6ODM*-T_{ADH1}-*attL2*	2
pCS2657	*attL1*-P_{TEF1}-*CODM*-T_{CYC1}-*attL2*	2
pCS2659	*attL1*-P_{CYC1}-*morA*-T_{PYK1}-*attL2*	2
pCS2660	*attL1*-P_{ADH1}-*odc1*-T_{GAP1}-*attL2*	2
pCS2661	*attL1*-P_{TPI1}-*cor1.3*-T_{STE2}-*attL2*	2
pCS2663	*attL1*-P_{PGK1}-*morB*-T_{PHO5}-*attL2*	2
pCS4187	Centromeric KanR, CRISPR construct for LEU2 locus insertion	3
pCS4305	Centromeric *URA3*; P_{GPD}-GFP-T_{ADH1}; P_{TEF1}-mCherry-T_{CYC1}	This work
pCS4306	Centromeric *URA3*; GFP- T_{ADH1}; P_{TEF1}-mCherry-T_{CYC1} (see Supp. Fig. 23)	This work
pCS4307	Centromeric *URA3*; P3-GFP- T_{ADH1}; P_{TEF1}-mCherry-T_{CYC1}	This work
pCS4308	Centromeric *URA3*; P4-GFP- T_{ADH1}; P_{TEF1}-mCherry-T_{CYC1}	This work
pCS4309	Centromeric *URA3*; P5-GFP- T_{ADH1}; P_{TEF1}-mCherry-T_{CYC1}	This work
pCS4339	kanR; P_{ACT1}-ZEV ATF-T_{CYC1}	This work

Supplementary Table 6: Plasmids used in this work.

**Strain**	**Genotype**
CSY3	W303 (*MATα, ade2–1; ura3–1; his3–11,15; trp1–1; leu2–3,112; can 1– 100*)
CSY1252	CSY3 *LEU2Δ*::P_{ACT1}-*ZEV*-T_{CYC1}

Supplementary Table 7: Yeast strains used in this work.

Name	Sequence	Purpose
pCS4339_pACT1-ZEV-backbone-fwd	TTGACGAGTACGGTGGGTAGctcgagtcatgtattagttatgtcacgcttacat	Cloning
pCS4339_backbone-pACT1-ZEV-rev	TGGGTCTGCAAGGTAGAGGCagcctgctttttgtacaaagttggcattataa	Cloning
pCS4339_backbone-pACT1-ZEV-fwd	TTTGTACAAAAAAGCAGGCTgcctctaccttgcagacccatataata	Cloning
pCS4339_pACT1-ZEV-backbone-rev	TAACTAATACATGACTCGAGctacccaccgtactcgtcaattccaag	Cloning
CSY1252_integration_F	AATTAGTGGGAGGTATTTACTTTGGTAAGAGAAAGGAAGAtgccaactttgtacaaaaaagcaggct	Cloning
CSY1252_integration_R	GGTGTATTGTTCACTATCCCAAGCGACACCATCACCATCGtgccaactttgtacaagaaagctgggt	Cloning
CSY1252_cPCR_5'_F	ccacaatttgctaaaggtactgacttcgttg	Cloning
CSY1252_cPCR_5'_R	gtgaggcatatgtttttaagggttttgaggatc	Cloning
CSY1252_cPCR_3'_F	ttggaattgacgagtacggtgggtag	Cloning
CSY1252_cPCR_3'_R	ggctcatgttgtagggccatgaaag	Cloning
pCS4305-6_pGPD_F	TGCGAAACGATCCTCATCCTGTCATCGATgagtttatcattatcaatactcgccatttc	Cloning
pCS4305_pGPD_R	GTGTTTATTCGAAACTAAGTTCTTGgtgttttaaaactaaaaaaaagactaactataaaagtagaat	Cloning
pCS4305_GFP_F	CAAGAACTTAGTTTCGAATAAACACACATAAACAAACAAAatgtctaaaggtgaagaattattcactgg	Cloning
pCS4305-6_GFP_R	CTGGTAACAAGACTGGACCATCACCAATTggagtattttgttgataatggtcagct	Cloning
pCS4306_pGPD-fragment_R	AGTGAATAATTCTTCACCTTTAGACGtcactactattaattatttacgtattctttgaaatgg	Cloning
pCS4306_GFP_F	gtctaaaggtgaagaattattcactggt	Cloning
pCS4306_P3_Gibs_F	TACGTAAATAATTAATAGTAGTGACttatattgaattttcaaaaattcttactttttttttggatg	Cloning
pCS4306_Pzev-all_Gibs_R	AGTGAATAATTCTTCACCTTTAGACATtgttaaggtagttcgttcggctctttgg	Cloning

pCS4306_P4_Gibs_F	AAGAATACGTAAATAATTAATAGTAGTGACttatattgaattttcaaaaattcttactttttt	Cloning
pCS4306_P8_Gibs_F	AAGAATACGTAAATAATTAATAGTAGTGACgcgtgggcgaattggtgcgt	Cloning
Gap_repair_Twist-designs_F	CGCCATTTCAAAGAATACGTAAATAATTAATAGTAGTGAC	Cloning
Gap_repair_Twist-designs_R	AATTGGGACAACACCAGTGAATAATTCTTCACCTTTAGACA	Cloning
Gap_repair_GPD_F	CGCCATTTCAAAGAATACGTAAATAATTAATAGTAGTGACgagtttatcattatcaatactcgccatttc	Cloning
Gap_repair_GPD_R	AATTGGGACAACACCAGTGAATAATTCTTCACCTTTAGACATtttgtttgtttatgtgtgtttattcgaaac	Cloning
Gap_repair_TEF_F	CGCCATTTCAAAGAATACGTAAATAATTAATAGTAGTGACatagcttcaaaatgtttctactcctttttac	Cloning
Gap_repair_TEF_R	AATTGGGACAACACCAGTGAATAATTCTTCACCTTTAGACATtatttcccgcggcaaaacttagatt	Cloning
Gap_repair_ADH1_F	CGCCATTTCAAAGAATACGTAAATAATTAATAGTAGTGACgcatgcaacttcttttcttttttttttctt	Cloning
Gap_repair_ADH1_R	AATTGGGACAACACCAGTGAATAATTCTTCACCTTTAGACATtgtataattgtatgcttggtatagcttgaa	Cloning
Gap_repair_PGK1_F	CGCCATTTCAAAGAATACGTAAATAATTAATAGTAGTGACaggcatttgcaagaattactcgt	Cloning
Gap_repair_PGK1_R	AATTGGGACAACACCAGTGAATAATTCTTCACCTTTAGACATtgtatatatttgttgtaaaaagtagataattacttccttg	Cloning
Gap_repair_TPI1_F	CGCCATTTCAAAGAATACGTAAATAATTAATAGTAGTGACcggaccttaatacattcagacacttc	Cloning
Gap_repair_TPI1_R	AATTGGGACAACACCAGTGAATAATTCTTCACCTTTAGACATtgtatattttagtttatgtatgtgtttttgtagttataga	Cloning
Gap_repair_CYC1_F	CGCCATTTCAAAGAATACGTAAATAATTAATAGTAGTGACgagcgttggttggtggatcaa	Cloning
Gap_repair_CYC1_R	AATTGGGACAACACCAGTGAATAATTCTTCACCTTTAGACATtgtatatattaatttagtgtgtgtatttgtgtttgt	Cloning

Gap_repair_ZEV_Pr3,4_F	CGCCATTTCAAAGAATACGTAAATAATTAATAGTAGTGACttatattgaattttcaaaaattcttacttttttttttggat	Cloning
Gap_repair_ZEV_R	AATTGGGACAACACCAGTGAATAATTCTTCACCTTTAGACATtgttaaggtagttcgttcggct	Cloning
Gap_repair_ZEV_Pr8_F	CGCCATTTCAAAGAATACGTAAATAATTAATAGTAGTGACgcgtgggcgaattggt	Cloning
sequencing_ZEV-ATF_M13F	gtaaaacgacggccagtcttaagc	Cloning
sequencing_ZEV-ATF_pACT1	cttactgcttttttcttcccaagatcgaaaatttactg	Cloning
sequencing_ZEV-ATF_gene-internal	gatgatgggcttactgaccaacctg	Cloning
sequencing_ZEV-ATF_tCYC1	cctagacttcaggttgtctaactccttcc	
sequencing_ZEV-ATF_M13R	agagctgccaggaaacagctatgac	Cloning
sequencing_pCS4305-9_GPD_F	catcttgttcaatcatgcgaaacgatcct	Cloning
sequencing_pCS4305-9_GPD_R	cttcaccggagacagaaaatttgtgacc	Cloning
sequencing_pCS4305_GFP_R	tggataaggcagattgagtggataagtaatg	Cloning
BK447	ctcaatggaGTGATGggtctcaGAAC	Library
BK448	tgctgggattctGTCCAGggtctcaGCTG	Library
BK450	ctcaatggaGTGATGggtctcaCAGC	Library
BK451	tgctgggattctGTCCAGggtctcaCCGT	Library
BK453	ctcaatggaGTGATGggtctcaACGG	Library
BK454	tgctgggattctGTCCAGggtctcaTGGT	Library
BK456	ctcaatggaGTGATGggtctcaACCA	Library
BK602	AATTGGGACAACACCAGTGAATAAT	Library
BK630	GTGATGggtctcaTGTCTAAAGGTGAAGAATTATTCACTGGTGTTGTCCCAATT	Library

BK631	tgctgggattctGTCCAGggtctcaGACA	Library
BK641	cgccatttcaaagaatacgtaaataattaatagtagtgacNNNNNNNNNNNNNNNNNNNNNNNNNNNNNNNNNNNNNNNNAGGTGTCTGGGTGAACtgagaccCTGGAC	Library
BK642	CGCCATTTCAAAGAATACGTAAATAATTAATAG	Library
BK643	tgctgggattctGTCCAGggtctcaGTTC	Library
BK645	GTGATGggtctcaCAGCACCAAAANNNNNNNNNNNNNNNNNNNNNNNNNNNNNNNNNNNNNNATAAGTATATAAAGACGGtgagaccCTGGAC	Library
BK646	GTGATGggtctcaACGG(N1:28090954)(N1)(N1)(N1)(N1)(N1)(N1)(N1)(N1)(N1)(N1)(N1)(N1)(N1)(N1)(N1)(N1)(N1)(N1)(N1)(N1)(N1)(N1)(N1)(N1)(N1)(N1)(N1)(N1)(N1)(N1)(N1)(N1)(N1)(N1)(N1)(N1)(N1)(N1)(N1)(N1)(N1)(N1)(N1)(N1)(N1)(N1)(N1)(N1)(N1)(N1)(N1)(N1)(N1)(N1)(N1)(N1)(N1)(N1)(N1)(N1)(N1)(N1)(N1)(N1)(N1)(N1)(N1)(N1)(N1)(N1)(N1)(N1)(N1)(N1)CACCAtgagaccCTGGAC	Library
BK660	ATGggtctcaGAACAGTT(N1:18180955)(N1)(N1)(N1)(N1)(N1)(N1)(N1)(N1)(N1)(N1)GGCATCCA(N2:30201634)(N2)(N2)(N2)(N2)(N2)(N2)(N2)(N2)(N2)(N2)(N2)(N2)(N2)(N2)(N2)(N2)(N2)(N2)(N2)(N2)(N2)(N2)(N2)(N2)GGCATCCA(N3:66111211)(N3)(N3)(N3)(N3)(N3)(N3)(N3)AGAATCCCAGCtgagaccCTG	Library
BK661	GTGATGggtctcaACCAAGHHHHHHHHHHHHHHHHHHHHHHHHHHHHHHHHHHHATGTCtgagaccCTGGAC	Library
BK675	cgccatttcaaagaatacgtaaataattaatagtagtgacNNNNNNNNNNNNNNNNNNNNNNNNNNNNNNNNNNNNNNNNTGTCTGGGTGtgagaccCTGGAC	Library
BK676	cgccatttcaaagaatacgtaaataattaatagtagtgacNNNNNNNNNNNNNNNNNNNNNNNNNNNNNNNNNNNNNNNNGGCAtgagaccCTGGAC	Library
BK677	ATGggtctcaGAACAGTTNNNNNNNNNNNGGCATCCANNNNNNNNNNNNNNNNNNNNNNNNNGGCATCCANNNNNNNNAGAATCCCAGCtgagaccCTG	Library

BK678	ATGggtctcaGGTGNNNNNNNNNNNGGCATCCANNNNNNNNNNNNNNNNNNNNNNNNNGGCATCCANNNNNNNNATCCCAGCCtgagaccCTG	Library
BK679	ATGggtctcaGGCATCCANNNNNNNNNNNNNNNNNNNNNNNNGGCATCCAtgagaccCTG	Library
BK680	ATGggtctcaAGCCANNNNNNNNNNNNNNNNNNNNNNNNNNNNNNNNNNNNATAAGTATATAAAGACGGtgagaccCTGGAC	Library
BK681	ATGggtctcaTCCANNNNNNNNNNNNNNNNNNNNNNNNNNNNNNNNNNNNATAAGTATATAAAGACGGtgagaccCTGGAC	Library
BK682	GTGATGggtctcaCAGCACCAAAANNNNNNNNNNNNNNNNNNNNNNNNNNNNNNNNNNNNNNNNNNNNNNNNNNNNNNNNNNNNNNNNNNNNNNNNNNNNNNNNNNATAAGTATATAAAGACGGtgagaccCTGGAC	Library
BK683	GTGATGggtctcaCAGCACCAAAANNNNNNNNNNNNNNNNNNNNNNNNNNNNNNNNNNNNNNNNNGTATATAAAGtgagaccCTGGAC	Library
BK684	GTGATGggtctcaTCCANNNNNNNNNNNNNNNNNNNNNNNNNNNNNNNNNNNNNNNNNNNNNNNNNNNNNNNNNNNNNNNNNNNNNNNNNNNNNGTATATAAAGtgagaccCTGGAC	Library
BK685	GTGATGggtctcaAAAG(N1:28090954)(N1)(N1)(N1)(N1)(N1)(N1)(N1)(N1)(N1)(N1)(N1)(N1)(N1)(N1)(N1)(N1)(N1)(N1)(N1)(N1)(N1)(N1)(N1)(N1)(N1)(N1)(N1)(N1)(N1)(N1)(N1)(N1)(N1)(N1)(N1)(N1)(N1)(N1)(N1)(N1)(N1)(N1)(N1)(N1)(N1)(N1)(N1)(N1)(N1)(N1)(N1)(N1)(N1)(N1)(N1)(N1)(N1)(N1)(N1)(N1)(N1)(N1)(N1)(N1)(N1)(N1)(N1)CACCAtgagaccCTGGAC	Library
BK688	cgccatttcaaagaatacgtaaataattaatagtagtgacNNNNNNNNNNNNNNNNNNNNNNNNNNNNNNNNNNNNNNNNGCGTtgagaccCTGGAC	Library
BK689	GTGATGggtctcagcgtgggcgNNNNNNNNNgcgtgggcgNgcgtgggcgNgcgtgggcgNgcgtgggcgtgagaccCTGGAC	Library
BK690	GTGATGggtctcagcgtgggcgNNNNNNNgcgtgggcgNNNNNNNNNgcgtgggcgtgagaccCTGGAC	Library

BK691	GTGATGggtctcaGGCGNNNNNNNNNNNNNNNNNNNNNNNNNNNNNNNNNNNNNATAAGTATATAAAGACGGtgagaccCTGGAC	Library
BK692	GTGATGggtctcaGGCGNNNNNNNNNNNNNNNNNNNNNNNNNNNNNNNNNNNNNNNNNNNNNNNNNNNNNNNNNNNNNNNNNNNNNNNNNNNNNNGTATATAAAGtgagaccCTGGAC	Library
BK693	GTCCAGggtctcaCACCCAG	Library
BK694	cgtGTCCAGggtctcaTGCC	Library
BK695	gacatcATGggtctcaGAACAGTT	Library
BK696	ATACAGggtctcaGCTGGGATTC	Library
BK697	cgtagcATGggtctcaGGTG	Library
BK698	TAACAGggtctcaGGCTGGGAT	Library
BK699	AGTATGggtctcaGGCATCCA	Library
BK700	TTACAGggtctcaTGGATGCC	Library
BK701	gcttaATGggtctcaAGCCA	Library
BK702	GTCCAGggtctcaCCGTCTT	Library
BK703	cgatgcATGggtctcaTCCA	Library
BK704	GTGATGggtctcaCAGCACCAAAA	Library
BK705	gctaGTCCAGggtctcaCTTTATATAC	Library
BK706	cgtaGTGATGggtctcaTCCA	Library
BK707	gacgtaGTGATGggtctcaAAAG	Library
BK708	gatGTCCAGggtctcaACGC	Library
BK709	GTGATGggtctcagcgtg	Library
BK710	GTCCAGggtctcacgccc	Library
BK711	catGTGATGggtctcaGGCG	Library
BK712	GTCCAGggtctcaCCGTCTT	Library
BK718	ATGggtctcaAGCCANNNNNNNNNNNNNNNNNNNNNNNNNNNNNNNNNNNNNNNNNNNNNNNNNNNNN	Library

	NNNNNNNNNNNNNNNNNNNNGTATATAAAGtgagaccCTGGAC	
BK1	CTTCACCGGAGACAGAAAATTTGTGACC	NGS Prep
BK445	CATCTTGTTCAATCATGCGAAACGATCCT	NGS Prep
BK719	AATGATACGGCGACCACCGAagttccGATCTACACTCTTTCCCTACACGACGCTCTTCCGATCTGAATACGTAAATAATTAATAGTAGTGAC	NGS Prep
BK720	AATGATACGGCGACCACCGAccgtccGATCTACACTCTTTCCCTACACGACGCTCTTCCGATCTttGAATACGTAAATAATTAATAGTAGTGAC	NGS Prep
BK721	AATGATACGGCGACCACCGAgtgaaaGATCTACACTCTTTCCCTACACGACGCTCTTCCGATCTccccGAATACGTAAATAATTAATAGTAGTGAC	NGS Prep
BK722	AATGATACGGCGACCACCGAgagtggGATCTACACTCTTTCCCTACACGACGCTCTTCCGATCTgacgacGAATACGTAAATAATTAATAGTAGTGAC	NGS Prep
BK723	AATGATACGGCGACCACCGAattcctGATCTACACTCTTTCCCTACACGACGCTCTTCCGATCTagatagatGAATACGTAAATAATTAATAGTAGTGAC	NGS Prep
BK724	CAAGCAGAAGACGGCATACGAGATcgtgatGTGACTGGAGTTCAGACGTGTGCTCTTCCGATCTTGAATAATTCTTCACCTTTAGACA	NGS Prep
BK725	CAAGCAGAAGACGGCATACGAGATtggtcaGTGACTGGAGTTCAGACGTGTGCTCTTCCGATCTaaTGAATAATTCTTCACCTTTAGACA	NGS Prep
BK726	CAAGCAGAAGACGGCATACGAGATgcctaaGTGACTGGAGTTCAGACGTGTGCTCTTCCGATCTatatTGAATAATTCTTCACCTTTAGACA	NGS Prep
BK727	CAAGCAGAAGACGGCATACGAGATattggcGTGACTGGAGTTCAGACGTGTGCTCTTCCGATCTccgccgTGAATAATTCTTCACCTTTAGACA	NGS Prep
BK728	CAAGCAGAAGACGGCATACGAGATgatctgGTGACTGGAGTTCAGACGTGTGCTCTTCCGATCTgcgctataTGAATAATTCTTCACCTTTAGACA	NGS Prep

BK729	CAAGCAGAAGACGGCATACGAGATaagctaGTGACTGGAGTTCAGACGTGTGCTCTTCCGATCTtgcacatgcaTGAATAATTCTTCACCTTTAGACA	NGS Prep
BK730	AATGATACGGCGACCACCGA	NGS Prep
BK731	CAAGCAGAAGACGGCATACG	NGS Prep
BK832	AATGATACGGCGACCACCGAGATCTACACaggctataACACTCTTTCCCTACACGACGCTCTTCCGATCTGAATACGTAAATAATTAATAGTAGTGAC	NGS Prep
BK833	AATGATACGGCGACCACCGAGATCTACACgcctctatACACTCTTTCCCTACACGACGCTCTTCCGATCTttGAATACGTAAATAATTAATAGTAGTGAC	NGS Prep
BK834	AATGATACGGCGACCACCGAGATCTACACaggataggACACTCTTTCCCTACACGACGCTCTTCCGATCTccccGAATACGTAAATAATTAATAGTAGTGAC	NGS Prep
BK835	AATGATACGGCGACCACCGAGATCTACACtcagagccACACTCTTTCCCTACACGACGCTCTTCCGATCTgacgacGAATACGTAAATAATTAATAGTAGTGAC	NGS Prep
BK836	AATGATACGGCGACCACCGAGATCTACACcttcgcctACACTCTTTCCCTACACGACGCTCTTCCGATCTagatagatGAATACGTAAATAATTAATAGTAGTGAC	NGS Prep
BK837	AATGATACGGCGACCACCGAGATCTACACtaagattaACACTCTTTCCCTACACGACGCTCTTCCGATCTgtgaagtgaaGAATACGTAAATAATTAATAGTAGTGAC	NGS Prep

Supplementary Table 8: Oligonucleotides used in this work. Purpose: Category of purpose the oligo was designed for: Cloning, Library, or NGS Prep. For Cloning oligos, uppercase bases are sequence homology for Gibson assembly or yeast gap repair; lowercase bases are homology for PCR. For Library oligos, all are single-stranded oligonucleotides, except BK630, which was ordered as a double-stranded fragment; lowercase bases indicate BsaI cut sites, annealing homology, or padding to raise PCR annealing temperatures. For NGS Prep oligos, lowercase bases denote barcode sequences.

Fragment #	Fragment Name	Template	Forward Primer	Reverse Primer
1	UAS_orig	BK641	BK642	BK643
2	TF_orig	BK660	BK447	BK448
3	Internal_orig	BK645	BK450	BK451
4	Core_orig	BK646	BK453	BK454
5	UTR_orig	BK661	BK456	BK631
6	UAS_Small/Unbiased	BK675	BK642	BK693
7	UAS_Neg/Unbiased	BK676	BK642	BK694
8	TF_Pos/Unbiased	BK677	BK695	BK696
9	TF_Small/Unbiased	BK678	BK697	BK698
10	TF_Neg/Unbiased	BK679	BK699	BK700
11	Int_Small/Normal/Pos	BK680	BK701	BK702
12	Int_Neg/Normal/Pos	BK681	BK703	BK702
13	Int_Pos/Extended/Pos	BK682	BK704	BK702
14	Int_Pos/Normal/Small	BK683	BK704	BK705
15	Int_Neg/Extended/Small	BK684	BK706	BK705
16	Core_Small/Normal	BK685	BK707	BK454
17	UAS_Neg/ZEV	BK688	BK642	BK708
18	TF_Neg/ZEV-1	BK689	BK709	BK710
19	TF_Neg/ZEV-2	BK690	BK709	BK710
20	Int_ZEV/Normal/Pos	BK691	BK711	BK712
21	Int_ZEV/Extended/Small	BK692	BK711	BK705
22	Int_Small/Extended/Small	BK718	BK701	BK705

Supplementary Table 9: Oligonucleotide combinations used in library fragment PCRs.

Condition	Gate #	Gate Lower Bound (GFP/mCherry, log10)	Gate Upper Bound (GFP/mCherry, log10)	FITC PMT voltage (V)	Cells Collected
Replicate A	1	NA	-0.39794001	483	1152086
Replicate A	2	-0.39794001	-0.30980392	483	877208
Replicate A	3	-0.30980392	-0.22184875	483	623617
Replicate A	4	-0.22184875	-0.13667714	483	1732474
Replicate A	5	-0.13667714	-0.04575749	483	2143602
Replicate A	6	-0.04575749	0.04139269	483	1459091
Replicate A	7	0.04139269	0.1271048	483	3343304
Replicate A	8	0.1271048	0.21484385	483	3321220
Replicate A	9	0.21484385	0.30103	483	1367478
Replicate A	10	0.30103	0.38916608	483	1454058
Replicate A	11	0.38916608	0.47712125	483	476187
Replicate A	12	0.47712125	NA	483	41897
Replicate B	1	NA	-0.39794001	483	1582011
Replicate B	2	-0.39794001	-0.30980392	483	1113797
Replicate B	3	-0.30980392	-0.22184875	483	1607476
Replicate B	4	-0.22184875	-0.13667714	483	2161085
Replicate B	5	-0.13667714	-0.04575749	483	2762150
Replicate B	6	-0.04575749	0.04139269	483	3678931
Replicate B	7	0.04139269	0.1271048	483	4191312
Replicate B	8	0.1271048	0.21484385	483	3971310
Replicate B	9	0.21484385	0.30103	483	3137650
Replicate B	10	0.30103	0.38916608	483	1677953
Replicate B	11	0.38916608	0.47712125	483	479008
Replicate B	12	0.47712125	NA	483	78768

Supplementary Table 10: Sort conditions in the P_{GPD} experiment.

**Condition**	**Gate #**	**Gate Lower Bound (GFP/mCherry, log10)**	**Gate Upper Bound (GFP/mCherry, log10)**	**FITC PMT voltage (V)**	**Cells Collected**
Uninduced	1	NA	-0.39794001	483	617000
Uninduced	2	-0.39794001	-0.30980392	483	662000
Uninduced	3	-0.30980392	-0.22184875	483	1390000
Uninduced	4	-0.22184875	-0.13667714	483	1702000
Uninduced	5	-0.13667714	-0.04575749	483	2390000
Uninduced	6	-0.04575749	0.04139269	483	3448000
Uninduced	7	0.04139269	0.1271048	483	3346000
Uninduced	8	0.1271048	0.21484385	483	3180000
Uninduced	9	0.21484385	0.30103	483	2226000
Uninduced	10	0.30103	0.38916608	483	1149000
Uninduced	11	0.38916608	0.47712125	483	224000
Uninduced	12	0.47712125	NA	483	16000
Induced	1	NA	-0.39794001	341	52000
Induced	2	-0.39794001	-0.30980392	341	57000
Induced	3	-0.30980392	-0.22184875	341	118000
Induced	4	-0.22184875	-0.13667714	341	174000
Induced	5	-0.13667714	-0.04575749	341	465000
Induced	6	-0.04575749	0.04139269	341	1176000
Induced	7	0.04139269	0.1271048	341	1823000
Induced	8	0.1271048	0.21484385	341	4643000
Induced	9	0.21484385	0.30103	341	2394000
Induced	10	0.30103	0.38916608	341	528000
Induced	11	0.38916608	0.47712125	341	20000
Induced	12	0.47712125	NA	341	1600

Supplementary Table 11: Sort conditions in the P_{ZEV} experiment.

Condition	Gate #	Gate Lower Bound (GFP/mCherry, log10)	Gate Upper Bound (GFP/mCherry, log10)	FITC PMT voltage (V)	Cells Collected
Uninduced	1	NA	-1.39794001	370	2302000
Uninduced	2	-1.39794001	-1.21043388	370	961000
Uninduced	3	-1.21043388	-1.02292776	370	1302000
Uninduced	4	-1.02292776	-0.83542163	370	1138000
Uninduced	5	-0.83542163	-0.6479155	370	697000
Uninduced	6	-0.6479155	-0.46040938	370	635000
Uninduced	7	-0.46040938	-0.27290325	370	1249000
Uninduced	8	-0.27290325	-0.08539712	370	833000
Uninduced	9	-0.08539712	0.102109	370	471000
Uninduced	10	0.102109	0.28961513	370	333000
Uninduced	11	0.28961513	0.47712125	370	75000
Uninduced	12	0.47712125	NA	370	1600
Induced	1	NA	-1.39794001	300	1150000
Induced	2	-1.39794001	-1.21043388	300	470000
Induced	3	-1.21043388	-1.02292776	300	731000
Induced	4	-1.02292776	-0.83542163	300	1443000
Induced	5	-0.83542163	-0.6479155	300	1115000
Induced	6	-0.6479155	-0.46040938	300	1393000
Induced	7	-0.46040938	-0.27290325	300	1988000
Induced	8	-0.27290325	-0.08539712	300	928000
Induced	9	-0.08539712	0.102109	300	536000
Induced	10	0.102109	0.28961513	300	625000
Induced	11	0.28961513	0.47712125	300	371000
Induced	12	0.47712125	NA	300	42400

Supplementary Table 12: Sort conditions in the design validation experiment.

Condition	Gate #	Cells Collected	Minipreps	Barcoding forward primer	Barcoding reverse primer
Replicate A	1	1152086	2	BK719	BK724
Replicate A	2	877208	1	BK720	BK724
Replicate A	3	623617	1	BK721	BK724
Replicate A	4	1732474	3	BK722	BK724
Replicate A	5	2143602	3	BK723	BK724
Replicate A	6	1459091	2	BK719	BK725
Replicate A	7	3343304	5	BK720	BK725
Replicate A	8	3321220	4	BK721	BK725
Replicate A	9	1367478	2	BK722	BK726
Replicate A	10	1454058	2	BK723	BK726
Replicate A	11	476187	1	BK719	BK726
Replicate A	12	41897	1	BK720	BK726
Replicate B	1	1582011	3	BK721	BK727
Replicate B	2	1113797	2	BK722	BK727
Replicate B	3	1607476	3	BK723	BK727
Replicate B	4	2161085	3	BK719	BK727
Replicate B	5	2762150	4	BK720	BK727
Replicate B	6	3678931	4	BK721	BK728
Replicate B	7	4191312	5	BK722	BK728
Replicate B	8	3971310	4	BK723	BK729
Replicate B	9	3137650	4	BK719	BK729
Replicate B	10	1677953	3	BK720	BK729
Replicate B	11	479008	1	BK721	BK729
Replicate B	12	78768	1	BK722	BK729

Supplementary Table 13: Oligonucleotides and miniprep counts in NGS prep for the P_{GPD} library.

**Condition**	**Gate #**	**Cells Collected**	**Minipreps**	**Barcoding forward primer**	**Barcoding reverse primer**
Uninduced	1	617000	1	BK832	BK724
Uninduced	2	662000	1	BK833	BK724
Uninduced	3	1390000	2	BK834	BK724
Uninduced	4	1702000	2	BK835	BK724
Uninduced	5	2390000	3	BK836	BK724
Uninduced	6	3448000	4	BK837	BK729
Uninduced	7	3346000	4	BK832	BK725
Uninduced	8-1	795000	1	BK833	BK725
Uninduced	8-2	2385000	3	BK834	BK725
Uninduced	9	2226000	3	BK835	BK729
Uninduced	10	1149000	2	BK836	BK729
Uninduced	11	224000	1	BK837	BK725
Uninduced	12	16000	1	BK832	BK726
Induced	1	52000	1	BK833	BK726
Induced	2	57000	1	BK834	BK727
Induced	3	118000	1	BK835	BK726
Induced	4	174000	1	BK836	BK726
Induced	5	465000	1	BK837	BK726
Induced	6	1176000	2	BK832	BK727
Induced	7	1823000	2	BK833	BK727
Induced	8-1	2902000	5	BK834	BK726
Induced	8-2	1741000	3	BK835	BK728
Induced	9	2394000	3	BK835	BK727
Induced	10	528000	1	BK836	BK727
Induced	11	20000	1	BK837	BK727
Induced	12	1600	1	BK832	BK728

Uninduced	Original Library	NA; prepare as for 3000000	3	BK836	BK728
Induced	Original Library	NA; prepare as for 3000000	3	BK837	BK728

Supplementary Table 14: Oligonucleotides and miniprep counts in NGS prep for the P_{ZEV} library. In this experiment, cells sorted into bin 8 of each replicate were prepared in two fractions.

Condition	Gate #	Cells Collected	Minipreps	Barcoding forward primer	Barcoding reverse primer
Uninduced	1	2302000	3	BK837	BK729
Uninduced	2	961000	1	BK833	BK724
Uninduced	3	1302000	2	BK834	BK724
Uninduced	4	1138000	2	BK835	BK724
Uninduced	5	697000	1	BK836	BK724
Uninduced	6	635000	1	BK832	BK725
Uninduced	7	1249000	2	BK833	BK725
Uninduced	8	833000	1	BK834	BK725
Uninduced	9	471000	1	BK835	BK725
Uninduced	10	333000	1	BK836	BK725
Uninduced	11	75000	1	BK832	BK726
Uninduced	12	1600	1	BK833	BK726
Induced	1	1150000	2	BK834	BK726
Induced	2	470000	1	BK835	BK726
Induced	3	731000	1	BK836	BK726
Induced	4	1443000	2	BK832	BK727
Induced	5	1115000	2	BK833	BK727
Induced	6	1393000	2	BK836	BK729
Induced	7	1988000	2	BK835	BK727
Induced	8	928000	1	BK837	BK728
Induced	9	536000	1	BK832	BK728
Induced	10	625000	1	BK833	BK728
Induced	11	371000	1	BK834	BK728
Induced	12	42400	1	BK835	BK728
Uninduced	Original Library	NA; prepare as for 500000	1	BK836	BK728

Induced	Original Library	NA; prepare as for 500000	1	BK832	BK729

Supplementary Table 15: Oligonucleotides and miniprep counts in NGS prep for the design validation FACS-seq.

**Experiment**	**NGS used for mean determination**	**5' barcode**	**5' skew**	**3' skew**	**3' barcode**	**Read threshold for inclusion**	**Replicate difference threshold for inclusion**
**P_{GPD}**	Nextseq 1x75	No	Yes	No	Yes	10	0.2
P_{ZEV}	Nextseq 1x75	Yes	Yes	No	Yes	20	NA
Validation	Miseq 2x300	Yes	Yes	Yes	Yes	10	NA

Supplementary Table 16: Bin assignment strategy for FACS-seq experiments. Read threshold for inclusion: minimum number of reads required in each replicate or condition.

Exper-iment	Condition	Mean min	Mean max	Mean density	Sigma min	Sigma max	Sigma density	Fuzz min	Fuzz max	Fuzz density	Sigma chosen	Fuzz chosen
P_{GPD}	Replicate A	-0.9	0.9	0.002	0.01	0.1	0.004	0.01	0.1	0.004	0.038	0.07
P_{GPD}	Replicate B	-0.9	0.9	0.002	0.01	0.1	0.004	0.01	0.1	0.004	0.042	0.07
P_{ZEV}	Uninduced	-0.9	0.9	0.002	0.01	0.1	0.004	0.01	0.1	0.004	0.046	0.062
P_{ZEV}	Induced	0.4	1.9	0.002	0.01	0.1	0.004	0.01	0.1	0.004	0.026	0.054
Valid-ation	Uninduced	-0.9	2	0.002	0.01	0.1	0.004	0.01	0.1	0.004	0.082	0.046
Valid-ation	Induced	-0.2	2.4	0.002	0.01	0.1	0.004	0.01	0.1	0.004	0.058	0.054

Supplementary Table 17: Mean fitting hyperparameter values in FACS-seq.

First cycle	Last cycle	# Mutations	Include parent sequence along with mutants?
1	50	20	No
51	100	10	No
101	150	3	No
151	200	1	No
201	250	1	Yes

Supplementary Table 18: Cycle-wise hyperparameter choices for all promoter sets designed using the evolution strategy.

First cycle	Last cycle	Gradient step	Normalization power
1	50	0.05	1
51	100	0.05	1
101	150	0.01	1.01
151	200	0.01	1.05
201	250	2.00E-03	1.1

Supplementary Table 19: Cycle-wise hyperparameter choices for all promoter sets designed using the gradient ascent strategy.

Supplementary Note 1: Reaction Conditions for PCR and Golden Gate Assembly.

PCR Amplification of Library Fragments

Randomized oligonucleotides were PCR-amplified to form library fragments (oligo combinations as described in Supplementary Table 9), using the KAPA HiFi PCR kit (KAPA Biosystems), at 50 uL scale, using 5 uL 100 nM oligo as template. The annealing temperature was 50 C; the extension time was 15 seconds; 5 cycles were run. PCR products were purified using the Zymo Research DNA Clean & Concentrate kit according to manufacturer's instructions.

Golden Gate Assembly of Libraries

15 fmol of each desired fragment to be assembled was combined in the following reaction mixture:

**Reagent**	**Volume (µL)**
Oligo BK630, 100 nM	1
10X T4 ligase buffer (NEB)	1
BsaI-HF (NEB)	0.5
T4 ligase (NEB)	0.5
Fragments	To 10 µL

Golden Gate reaction products were PCR-amplified using KAPA HiFi PCR, at 4x100 uL scale, using 1 µL raw product as template in each reaction, and using BK602 and BK642 as primers. The annealing temperature was 55 C; the extension time was 30 seconds; 7 cycles were run. PCR products were purified using the Zymo Research DNA Clean & Concentrate kit according to manufacturer's instructions, eluting to 30 µL (total volume); 10 µL of this (total) was used as template at 6x100 uL scale. These reactions ran for 8 cycles, but otherwise used the same parameters. Final products were cleaned up using the Clean & Concentrate kit, according to manufacturer's instructions, and concentrated by Spin-Vac, yielding 600-800 ng insert DNA.

DNA recovery and NGS prep post-FACS

FACS-sorted cells were allowed to grow to stationary phase in YNB-U media with shaking at 30 C. 1.5-mL aliquots of cell culture were used as input in minipreps with the Zymoprep Yeast Plasmid Miniprep II kit (Zymo Research), according to manufacturer's instructions, as shown in Supplementary Tables 13-15. Minipreps were PCR-amplified using KAPA HiFi PCR, at 100 uL scale, using the entire miniprep volume as template in each reaction, and using BK1 and BK445 as primers. The annealing temperature was 60 C; the extension time was 30 seconds; 10 cycles were run. 10 uL of this product was then used as template in the same reaction conditions, and run for 6 cycles.

Barcoding PCR was carried out using oligos shown in Supplementary Tables 13-15, using KAPA HiFi PCR, at 80 uL scale, using 8 µL raw PCR product from the previous step as template. The annealing temperature was 50 C; the extension time was 30 seconds; 7 cycles were run. The products were cleaned up using the Clean & Concentrate kit according to manufacturer's jnstructions, quantitated by Qubit, mixed down to achieve an equal (DNA concentration/original # cells collected) ratio for each bin (except that Bins 12 were overrepresented tenfold to achieve coverage for rare sequences), and gel-extracted on a 2% agarose gel with SybrSafe Red dye.

The entire gel-extracted volume was used as template in a final 100 uL KAPA HiFi PCR, using BK730 and BK731 as primers. The annealing temperature was 55 C; the extension time was 30 seconds; 6 cycles were run. The product was cleaned up using the Clean & Concentrate kit according to manufacturer's instructions, tested using Qubit and Bioanalyzer as described in the main text, and submitted for NGS.

Supplementary Note 2: P_{GPD} sequence definition and expression context.

As described in the main text, a P_{GPD} sequence previously reported for use in metabolic engineering applications[2] was PCR-amplified from pCS2656. This sequence was revised to restore the wild-type yeast 5' UTR, which had been modified to include sites for restriction cloning.

The resulting 675-bp sequence was divided into a 60-bp upstream context sequence and a 615-bp sequence defined as the P_{GPD} promoter throughout the text; the full 675-bp sequence appears below.

>pGPD_675

```
GAGTTTATCATTATCAATACTCGCCATTTCAAAGAATACGTAAATAATTAATAGTAGTGATTTTCCTAACTTTATTT
AGTCAAAGAATTAGCCTTTTAATTCTGCTGTAACCCGTACATGCCCAAAATAGGGGGCGGGTTACACAGAATATATA
ACATCGTAGGTGTCTGGGTGAACAGTTTATTCCTGGCATCCACTAAATATAATGGAGCCCGCTTTTTAAGCTGGCAT
CCAGAAAAAAAAAGAATCCCAGCACCAAAATATTGTTTTCTTCACCAACCATCAGTTCATAGGTCCATTCTCTTAGC
GCAACTACAGAGAACAGGGGCACAAACAGGCAAAAAACGGGCACAACCTCAATGGAGTGATGCAACCTGCCTGGAGT
AAATGATGACACAAGGCAATTGACCCACGCATGTATCTATCTCATTTTCTTACACCTTCTATTACCTTCTGCTCTCT
CTGATTTGGAAAAAGCTGAAAAAAAAGGTTGAAACCAGTTCCCTGAAATTATTCCCCTACTTGACTAATAAGTATAT
AAAGACGGTAGGTATTGATTGTAATTCTGTAAATCTATTTCTTAAACTTCTTAAATTCTACTTTTATAGTTAGTCTT
TTTTTTAGTTTTAAAACACCAAGAACTTAGTTTCGAATAAACACACATAAACAAACAAA
```

Supplementary References

1. Liang, J. C., Chang, A. L., Kennedy, A. B. & Smolke, C. D. A high-throughput, quantitative cell-based screen for efficient tailoring of RNA device activity. *Nucleic Acids Res.* **40**, e154 (2012).

2. Thodey, K., Galanie, S. & Smolke, C. D. A microbial biomanufacturing platform for natural and semisynthetic opioids. *Nat. Chem. Biol.* **10**, 837–44 (2014).

3. Kotopka, B. J. & Smolke, C. D. Production of the cyanogenic glycoside dhurrin in yeast. *Metab. Eng. Commun.* **9**, e00092 (2019).
