# Supplementary material for: Model-driven generation of artificial yeast promoters
Source: Nat Commun. 2020 Apr 30;11:2113. doi: 10.1038/s41467-020-15977-4 (PMC7192914; doi:10.1038/s41467-020-15977-4)
Supplement: Supplementary file 2 — Reporting Summary [file 41467_2020_15977_MOESM2_ESM.pdf]

nature research

Corresponding author(s): Christina D. Smolke

Last updated by author(s): Mar 31, 2020

Reporting Summary

Nature Research wishes to improve the reproducibility of the work that we publish. This form provides structure for consistency and transparency in reporting. For further information on Nature Research policies, see Authors & Referees and the Editorial Policy Checklist .

Statistics

For all statistical analyses, confirm that the following items are present in the figure legend, table legend, main text, or Methods section.

n/a	Confirmed	
☐	☒	The exact sample size (*n*) for each experimental group/condition, given as a discrete number and unit of measurement
☐	☒	A statement on whether measurements were taken from distinct samples or whether the same sample was measured repeatedly
☐	☒	The statistical test(s) used AND whether they are one- or two-sided<br>*Only common tests should be described solely by name; describe more complex techniques in the Methods section.*
☒	☐	A description of all covariates tested
☐	☒	A description of any assumptions or corrections, such as tests of normality and adjustment for multiple comparisons
☐	☒	A full description of the statistical parameters including central tendency (e.g. means) or other basic estimates (e.g. regression coefficient) AND variation (e.g. standard deviation) or associated estimates of uncertainty (e.g. confidence intervals)
☐	☒	For null hypothesis testing, the test statistic (e.g. *F*, *t*, *r*) with confidence intervals, effect sizes, degrees of freedom and *P* value noted<br>*Give P values as exact values whenever suitable.*
☒	☐	For Bayesian analysis, information on the choice of priors and Markov chain Monte Carlo settings
☒	☐	For hierarchical and complex designs, identification of the appropriate level for tests and full reporting of outcomes
☐	☒	Estimates of effect sizes (e.g. Cohen's *d*, Pearson's *r*), indicating how they were calculated

Our web collection on statistics for biologists contains articles on many of the points above.

Software and code

Policy information about availability of computer code

Data collection	Flow cytometry: FACSDiva 8.0.1; Next-generation sequencing: Illumina MiSeq Control Software v3.0, NextSeq Control Software v2.1.0
Data analysis	Commercial code: Illumina bcl2fastq2 2.20, Windows 10, RStudio 1.1.463<br>Open source code: Paired-End reAd mergeR (PEAR) 0.9.6, Ubuntu 16.04, Python 2.7, R 3.5.1. Needleman-Wunsch CUDA implementation (unversioned: link to correct Git commit provided instead - https://github.com/hgbrian/nw_align/tree/54221ee)<br><br>Custom code: available at https://github.com/smolkelab/promoter_design (see Code availability)

For manuscripts utilizing custom algorithms or software that are central to the research but not yet described in published literature, software must be made available to editors/reviewers. We strongly encourage code deposition in a community repository (e.g. GitHub). See the Nature Research guidelines for submitting code & software for further information.

Data

Policy information about availability of data

All manuscripts must include a data availability statement. This statement should provide the following information, where applicable:
- Accession codes, unique identifiers, or web links for publicly available datasets
- A list of figures that have associated raw data
- A description of any restrictions on data availability

NGS data that support the conclusions of this study have been deposited in the NCBI Gene Expression Omnibus (GEO) with the accession code GSE135464 ([https://www.ncbi.nlm.nih.gov/geo/query/acc.cgi?acc=GSE135464]). All other data is available in Zenodo ([https://doi.org/10.5281/zenodo.3735426]), including R scripts and raw data for regenerating all data figures. The source data underlying Figs 3, 4, 5c-e and Supplementary Figs 15-18, 20, and 21 are provided as a Source Data

file.

Field-specific reporting

Please select the one below that is the best fit for your research. If you are not sure, read the appropriate sections before making your selection.

☒ Life sciences ☐ Behavioural & social sciences ☐ Ecological, evolutionary & environmental sciences

For a reference copy of the document with all sections, see nature.com/documents/nr-reporting-summary-flat.pdf

Life sciences study design

All studies must disclose on these points even when the disclosure is negative.

Sample size	Sample sizes were not statistically predetermined, but instead set based on the throughput of the assays and DNA synthesis methods used. Sample sizes for the initial FACS-seq experiments (~10^5) were sufficient to yield robust models (Figs. 2, S8). Sample size for other experiments was determined to be adequate based on the magnitude and consistency of measurable differences between groups.
Data exclusions	No data were excluded from the analyses.
Replication	The pGPD FACS-seq experiment was carried out in duplicate, and results from each replicate were very strongly correlated (Fig. S7). Representative sequences from the pZEV FACS-seq experiment were retested in the validation FACS-seq experiment (Fig. S13 "ZEV-grid" and S14), and designed sequences were further tested in single-sequence flow cytometry validation (Fig. 4A). All attempts to verify reproducibility were successful.
Randomization	Randomization was not relevant to this study, as the biological subjects for assays were bulk samples of microbial cells.
Blinding	Blinding was not relevant to this study, as the biological subjects for assays were bulk samples of microbial cells.

Reporting for specific materials, systems and methods

We require information from authors about some types of materials, experimental systems and methods used in many studies. Here, indicate whether each material, system or method listed is relevant to your study. If you are not sure if a list item applies to your research, read the appropriate section before selecting a response.

Materials & experimental systems

n/a	Involved in the study	
☒	☐	Antibodies
☐	☒	Eukaryotic cell lines
☒	☐	Palaeontology
☒	☐	Animals and other organisms
☒	☐	Human research participants
☒	☐	Clinical data

Methods

n/a	Involved in the study	
☒	☐	ChIP-seq
☐	☒	Flow cytometry
☒	☐	MRI-based neuroimaging

Eukaryotic cell lines

Policy information about cell lines

Cell line source(s)	Wild-type Saccharomyces cerevisiae strains CEN.PK2-1D and W303 were obtained from EuroSCARF (30000B; 20000A respectively)
Authentication	Cell lines were not independently authenticated by the authors.
Mycoplasma contamination	Yeast cell lines were not tested for mycoplasma contamination.
Commonly misidentified lines (See ICLAC register)	No commonly misidentified cell lines were used in this study.

Flow Cytometry

Plots

Confirm that:

- [x] The axis labels state the marker and fluorochrome used (e.g. CD4-FITC).
- [x] The axis scales are clearly visible. Include numbers along axes only for bottom left plot of group (a 'group' is an analysis of identical markers).
- [x] All plots are contour plots with outliers or pseudocolor plots.
- [x] A numerical value for number of cells or percentage (with statistics) is provided.

Methodology

Sample preparation	Yeast cultures were grown in yeast nitrogen base (BD Diagnostics) + 2% dextrose media lacking uracil, and passaged at least three times before sorting, with at least 10 OD600*mL units transferred in each passage. For experiments involving PZEV promoters, separate cultures with and without 1 µM beta-estradiol added were started 18 hours before the sort. Cultures were back-diluted to an OD of 0.05-0.1 5 hours before the sort to maintain them in log phase.<br><br>Cultures were harvested at an OD600 of 0.7-0.8, spun down, and resuspended in phosphate-buffered saline (PBS) with 10 µg/mL DAPI (ThermoFisher).
Instrument	FACSAria II (BD Biosciences); MACSQuant VYB (Miltenyi Biotec GmbH)
Software	Collection: FACSDiva; analysis: flowCore 1.48.1 (installed via Bioconductor)
Cell population abundance	Cell population abundances are given in Supplementary Tables 10-12. Because promoter activity can vary based on culture conditions, sample purity was not tested after sorting.
Gating strategy	FSC/SSC, singlet, DAPI, mCherry, and GFP gates were used to select viable, construct-expressing yeast. Gating parameters:<br>FSC/SSC: FSC-A > 20000, FSC-A < 240000<br>Singlet: FSC-H > 0.7*FSC-A + 4000<br>DAPI: log10(SSC-A) > 1.19*log10(Pacific-Blue-A) - 0.86<br>mCherry: mCherry-A > 500, mCherry-A < 30000<br>GFP: FITC-A > 20<br>Cells passing all these gates are GFP-positive; others are negative.

- [x] Tick this box to confirm that a figure exemplifying the gating strategy is provided in the Supplementary Information.
